# A rice fungal MAMP-responsive MAPK cascade regulates metabolic flow to antimicrobial metabolite synthesis

**DOI:** 10.1111/j.1365-313X.2010.04264.x

**Published:** 2010-06-21

**Authors:** Mitsuko Kishi-Kaboshi, Kazunori Okada, Leona Kurimoto, Shinya Murakami, Toshiaki Umezawa, Naoto Shibuya, Hisakazu Yamane, Akio Miyao, Hiroshi Takatsuji, Akira Takahashi, Hirohiko Hirochika

**Affiliations:** 1 Division of Plant Sciences, National Institute of Agrobiological SciencesTsukuba, Ibaraki 305-8602, Japan; 2 Biotechnology Research Center, the University of TokyoBunkyo-ku, Tokyo, 113-8657, Japan; 3 Department of Life Sciences, Faculty of Agriculture, Meiji UniversityKawasaki, Kanagawa 214-8571, Japan; 4 Research Institute for Sustainable Humanosphere, Kyoto UniversityGokasho, Uji, Kyoto 611-0011, Japan; 5 Division of Genome and Biodiversity Research, National Institute of Agrobiological SciencesTsukuba, Ibaraki 305-8602, Japan

**Keywords:** MAPK cascade, phytoalexin, rice, gene expression, MAMP

## Abstract

Plants recognize potential microbial pathogens through microbial-associated molecular patterns (MAMPs) and activate a series of defense responses, including cell death and the production of reactive oxygen species (ROS) and diverse anti-microbial secondary metabolites. Mitogen-activated protein kinase (MAPK) cascades are known to play a pivotal role in mediating MAMP signals; however, the signaling pathway from a MAPK cascade to the activation of defense responses is poorly understood. Here, we found in rice that the chitin elicitor, a fungal MAMP, activates two rice MAPKs (OsMPK3 and OsMPK6) and one MAPK kinase (OsMKK4). OsMPK6 was essential for the chitin elicitor-induced biosynthesis of diterpenoid phytoalexins. Conditional expression of the active form of OsMKK4 (OsMKK4^DD^) induced extensive alterations in gene expression, which implied dynamic changes of metabolic flow from glycolysis to secondary metabolite biosynthesis while suppressing basic cellular activities such as translation and cell division. OsMKK4^DD^ also induced various defense responses, such as cell death, biosynthesis of diterpenoid phytoalexins and lignin but not generation of extracellular ROS. OsMKK4^DD^-induced cell death and expression of diterpenoid phytoalexin pathway genes, but not that of phenylpropanoid pathway genes, were dependent on OsMPK6. Collectively, the OsMKK4–OsMPK6 cascade plays a crucial role in reprogramming plant metabolism during MAMP-triggered defense responses.

## Introduction

Plants are able to sense the presence of microbial organisms and initiate defense responses at the level of each single cell, and use two distinct defense systems to recognize and respond to pathogen challenges. A first defense system is triggered by recognition of microbe-associated molecular patterns (MAMPs), termed MAMP-triggered innate immunity (MTI). MTI includes activation of mitogen-activated protein kinase (MAPK), production of reactive oxygen species (ROS) and activation of transcription factors (Ausubel, 2005). Successful pathogens can deliver effectors that suppress the innate immune responses and contribute to their virulence (Chisholm *et al.*, 2006; Jones and Dangl, 2006). The second defense system is effector-triggered immunity (ETI), which occurs after recognition of the pathogen effectors by host resistance proteins. ETI triggers rapid defense responses that often include local programmed cell death, known as the hypersensitive response (HR) (Nimchuk *et al.*, 2003). Both MTI and ETI include similar processes that result from accumulation of ROS, transcriptional activation of pathogenesis-related genes, synthesis of antimicrobial secondary metabolites and cell-wall reinforcement via the oxidative cross-linking of cell-wall components and the deposition of lignins (Nurnberger *et al.*, 2004). However, most of these observations have been made in dicot plants such as Arabidopsis and tobacco and little is known for rice (*Oryza sativa*).

Phytoalexins are defined as low-molecular-weight antimicrobial compounds that are produced by plants in response to pathogens (Hammerschmidt, 1999; Dixon, 2001). A wide variety of different secondary metabolites serve as phytoalexins in various plant species. Rice accumulates multiple labdane-related diterpenoid phytoalexins in leaves after infection by a blast fungus, *Magnaporthe oryzae* (Cartwright *et al.*, 1981; Peters, 2006). Geranylgeranyl diphosphate (GGDP) is a common precursor in the biosynthetic pathway for these diterpenoids and for gibberellin (GA). Geranylgeranyl diphosphate is converted to *ent*-kaurene by the action of two diterpene cyclases, *ent*-copalyl diphosphate synthase (CPS) and *ent*-kaurene synthase (KS). Arabidopsis contains only one set of *CPS* and *KS* genes for GA biosynthesis (Yamaguchi, 2008). In contrast, rice contains additional sets of *CPS* and *KS* genes for phytoalexin biosynthesis (Prisic *et al.*, 2004; Sakamoto *et al.*, 2004). Chitin elicitor induces the accumulation of diterpenoid phytoalexins in association with expression of their biosynthetic enzyme genes (Yamada *et al.*, 1993; Okada *et al.*, 2007; Shimura *et al.*, 2007). OsTGAP1 was found to regulate the biosynthesis of a class of diterpenoid phytoalexins in response to the chitin elicitor signal (Okada *et al.*, 2009). However, the signaling pathway leading to the activation of these genes is poorly understood.

Phenylpropanoids, including lignin, flavonoids and many small molecules, are synthesized during pathogen infection and used for defensive functions across plant species. During defense responses, lignin and lignin-like phenolic compounds accumulate throughout HR regions in many plants (Campbell and Ellis, 1992; Lange *et al.*, 1995; Hano *et al.*, 2006; Menden *et al.*, 2007). The lignin content also increases in rice leaves infected with *M. oryzae* (Cai *et al.*, 2008). The structure of an inducible defense lignin is different from that of developmentally accumulated lignins (Lange *et al.*, 1995). It is believed that inducible defense lignin serves as a physical barrier against the spread of pathogen infection (Moerschbacher *et al.*, 1990; Franke *et al.*, 2002). The expression of several genes encoding phenylpropanoid biosynthetic enzymes is induced during treatment with chitin elicitor (Kaku *et al.*, 2006), but the signaling pathway from MAMPs to lignin biosynthesis remains largely unknown.

Mitogen-activated protein kinase (MAPK) cascades consist of kinase signaling modules that are evolutionarily conserved throughout eukaryotes (Ichimura *et al.*, 2002). A MAPK cascade minimally consists of three kinases: a MAPK, a MAPK kinase (MAPKK) and a MAPKK kinase (MAPKKK). MAPK cascades have important functions in regulating stress responses (Nakagami *et al.*, 2005; Pedley and Martin, 2005). Studies in Arabidopsis have shown three MAPKs, AtMPK3, AtMPK4 and AtMPK6, that become activated in response to MAMPs (Asai *et al.*, 2002; Andreasson *et al.*, 2005). AtMPK3 and AtMPK6 share common upstream MAPKKs, AtMKK4 and AtMKK5 (Asai *et al.*, 2002; Ren *et al.*, 2002). In Arabidopsis this MAPK cascade regulates cell death and the production of ROS and an indole-derived phytoalexin, camalexin (Ren *et al.*, 2002, 2008). There are 15 MAPK and eight MAPKK genes in rice (Hamel *et al.*, 2006). The rice proteins OsMPK3 (previously named as OsMAPK5 in Xiong and Yang, 2003), OsMPK4 and OsMPK6 (previously named as OsMPK2 in Kurusu *et al.*, 2005; or OsMAPK6 in Lieberherr *et al.*, 2005) are closely related to AtMPK3, AtMPK4 and AtMPK6, respectively. It has been reported that OsMPK6 is activated by several MAMPs (Kurusu *et al.*, 2005; Lieberherr *et al.*, 2005) and OsMPK3 by blast infection (Xiong and Yang, 2003); however, their upstream MAPKKs and downstream events have remained elusive.

In this study, we demonstrated that a rice MAPK cascade (OsMKK4–OsMPK3/OsMPK6) mediates a fungal chitin elicitor signal and regulates defensive responses including antimicrobial biosynthesis leading to plant cell death without extracellular ROS production. We also demonstrated, using genome-wide gene expression analysis, that the MAPK cascade positively regulates the genes for glycolysis and those for the biosynthesis of rice-specific diterpenoid phytoalexins and antimicrobial lignin, leading to actual accumulation of these antimicrobial compounds. This cascade also shows both positive and negative regulation of genes involved in various other cellular processes that promote defense responses.

## Results

### A MAPK pathway mediates an elicitor signal resulting in diterpenoid phytoalexin biosynthesis

**Figure 1:**
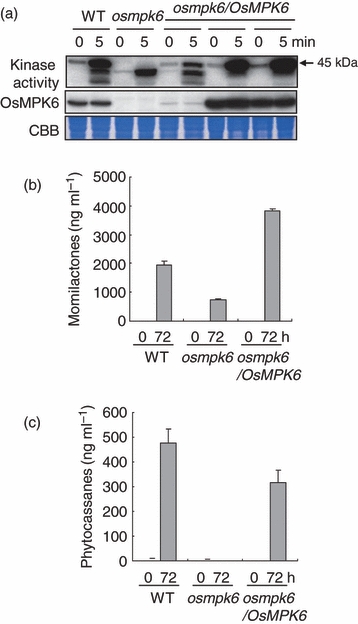
Activation of mitogen-activated protein kinase (MAPK) and accumulation of phytoalexins by treatment with chitin elicitor.Wild-type (WT), *osmpk6* and *osmpk6/OsMPK6* suspension cells were treated with chitin elicitor (1 μg/ml) or a solution without elicitor (mock) and harvested at the indicated times.(a) In-gel kinase assays after chitin elicitor treatment: WT, *osmpk6* and *osmpk6*/*OsMPK6* cells were tested. Kinase activities were analyzed using in-gel kinase assays with myelin basic protein (MBP) as a substrate (top panel). The arrow indicates the position of OsMPK6 with a size of 45 kDa. The OsMPK6 protein was detected by immunoblot analysis (middle panel). Equal loading was confirmed by CBB staining (bottom panel).(b), (c) The amounts of momilactone A and B (b) and phytocassane A–E (c) in rice cell culture media, as determined by high-performance liquid chromatography–electrospray tandem mass spectrometry (HPLC–ESI-MS/MS). Two independent cell lines for each genotype were used and three independent experiments were performed for each line with reproducible results. The bars represent means with standard deviations (*n*=3).

In Arabidopsis, MAMPs such as the conserved domain of flagellin (flg22) and the chitin elicitor activate AtMPK3 and AtMPK6 (Asai *et al.*, 2002; Miya *et al.*, 2007). To examine whether the chitin elicitor activates a MAPK cascade in rice, we performed an in-gel kinase assay using cultured rice cells treated with the elicitor. The chitin elicitor immediately and transiently activated numerous kinases (Figure S1 in Supporting Information). We then raised specific antibodies against OsMPK3, OsMPK4 and OsMPK6 (Figure S2). Immunoprecipitation (IP) kinase assays using these antibodies revealed that chitin elicitor activates OsMPK3, OsMPK4 and OsMPK6 (Figure S3). We analyzed *osmpk6* knockout mutant rice cells (Kurusu *et al.*, 2005), which fail to express *OsMPK6* transcripts due to the insertion of a retrotransposon *Tos17* in the fourth exon of the *OsMPK6* gene (Figure S4). An in-gel kinase assay (Figure 1a) showed that the 45-kDa band was absent in the *osmpk6* mutant cells but present in the *osmpk6*/*OsMPK6* cells, which harbor *OsMPK6* cDNA fused with its upstream promoter sequence in the *osmpk6* mutant background, after treatment with the chitin elicitor. Thus, the 45-kDa band was indeed OsMPK6. Other bands below OsMPK6 were seen in wild-type (WT), *osmpk6* and *osmpk6/OsMPK6* cells after treatment with the elicitor, and are therefore likely to be due to other MAPKs, presumably including OsMPK3 and OsMPK4.

In rice, diterpenoid phytoalexins are synthesized and accumulate after exposure to microorganisms (Peters, 2006). We analyzed the accumulation of momilactones and phytocassanes in the culture media of elicitor-treated WT, *osmpk6* and *osmpk6*/*OsMPK6* rice cells using high-performance liquid chromatography–electrospray tandem mass spectrometry (HPLC–ESI-MS/MS). Both kinds of phytoalexin were clearly accumulated in WT cells after elicitor treatment, but their levels were significantly reduced in the *osmpk6* mutant (Figure 1b,c). In the *osmpk6*/*OsMPK6* cells, which accumulated OsMPK6 protein at a similar level to that in WT cells (Figure S5), the levels of momilactones and phytocassanes were recovered. These results indicate that the MAPK pathway mediates MAMP signaling leading to diterpenoid phytoalexin biosynthesis, and that OsMPK6 is a crucial component of this signaling cascade.

### OsMKK4 is an upstream kinase for OsMPK3 and OsMPK6, and is activated by the chitin elicitor

To elucidate the MAPK cascade that mediates the chitin elicitor signal, we attempted to identify an upstream kinase that activates OsMPK6. OsMKK4 and OsMKK5 belong to the group C MAPKKs (Hamel *et al.*, 2006) and are closely related to AtMKK4, AtMKK5 and tobacco NtMEK2 (Figure S6). The *OsMKK5* gene contains two alternatively spliced isoforms according to the Rice Annotation Project (RAP) database (http://rapdb.dna.affrc.go.jp/). Since we could amplify only the longer sequence (corresponding to cDNA AK099769) by RT-PCR (data not shown), we analyzed only this longer sequence further. We generated constitutively active forms of the MAPKKs (OsMKK4^DD^ and OsMKK5^DD^) by mutating the conserved Ser/Thr in the activation loop [(S/T)XXXXX(S/T)] to Asp (Yang *et al.*, 2001). An *in vitro* kinase assay using recombinant proteins revealed that both OsMKK4^WT^ and OsMKK4^DD^ clearly activate OsMPK3 and OsMPK6 (Figure S6). OsMPK4 was also activated by OsMKK4^WT^ and OsMKK4^DD^, but only weakly compared with OsMPK3 and OsMPK6, suggesting that OsMPK4 is not a cognate target of OsMKK4. OsMKK5^WT^ showed weaker activity than both OsMKK4^WT^ and OsMKK4^DD^. OsMKK5^DD^ activated OsMPK6 but not OsMPK3 or OsMPK4 *in vitro*.

**Figure 2:**
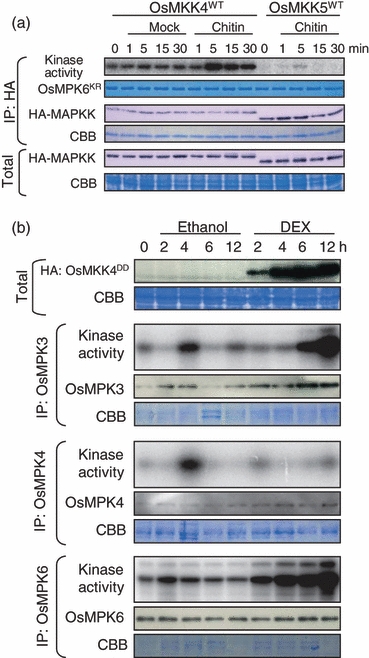
Chitin elicitor activates OsMKK4 and constitutive active OsMKK4 activates OsMPK3 and OsMPK6 *in vivo*.(a) Cells constitutively expressing hemagglutinin (HA) epitope-tagged mitogen-activated protein kinase kinase (MAPKK) in the wild-type (WT) form were treated with chitin elicitor (Chitin). The MAPKKs were immunoprecipitated and kinase activities were determined by immunoprecipitation (IP) kinase assays using kinase-inactive OsMPK6 (OsMPK6^KR^) as a substrate. Phosphorylation of OsMPK6^KR^ was detected by autoradiography after SDS-PAGE (top panel). Production of the MAPKKs was detected by immunoblot analysis using the anti-HA antibody (third and fifth panels). Equal loading was confirmed by CBB staining (second, fourth and bottom panels).(b) Myelin basic protein (MBP) kinase activities of OsMPK3, OsMPK4 and OsMPK6 in lines expressing *OsMKK4*^*DD*^ after induction by dexamethasone (DEX). Production of OsMKK4^DD^ was confirmed by immunoblot analysis using the anti-HA antibody. Activation of OsMPK3, OsMPK4 and OsMPK6 was determined using an IP kinase assay. The kinases were immunoprecipitated from 10 μg (OsMPK6), 50 μg (OsMPK3) or 100 μg (OsMPK4) of total proteins using specific antibodies. Phosphorylation of MBP was detected by autoradiography after SDS-PAGE (each top panel). Mitogen-activated protein kinases (MAPKs) were detected by immunoblot analysis (each middle panel). Equal loading was confirmed by CBB staining (each bottom panel).

To examine whether OsMKK4 and OsMKK5 are activated by MAMPs, we used transgenic rice cells expressing hemagglutinin (HA)-tagged *OsMKK4* and *OsMKK5*. Activation of OsMKK4 and OsMKK5 was determined by an IP kinase assay after chitin elicitor treatment. After immunoprecipitation, the kinase activities of the MAPKK proteins were measured using an inactive form of OsMPK6 (OsMPK6^KR^), which lacks the ATP-binding site, as a substrate. OsMKK4 showed a basal activity in phosphorylating OsMPK6^KR^, and chitin elicitor treatment increased this activity within 5 min (Figure 2a). OsMKK5 was also activated by elicitor treatment, but had less activity than OsMKK4.

Next, to investigate whether OsMKK4 and OsMKK5 activate MAPKs *in vivo*, we generated transgenic rice cell lines expressing HA-tagged *OsMKK4*^*WT*^, *OsMKK4*^*DD*^, *OsMKK5*^*WT*^ and *OsMKK5*^*DD*^ under the control of a dexamethasone (DEX)-inducible promoter. All of the transgene-derived products accumulated within 2 h of the beginning of DEX treatment (Figure S7). An endogenous kinase of 45 kDa, which corresponds to the size of OsMPK6, was activated only in the cells expressing *OsMKK4*^*DD*^. We then analyzed the activity of OsMPK6, OsMPK3 and OsMPK4 by an IP kinase assay in cells transformed with the MAPKK constructs and treated with DEX (Figure 2b). OsMPK6 and OsMPK3 were activated within 2 and 6 h of DEX treatment, indicating that OsMKK4 is an upstream kinase of OsMPK3 and OsMPK6. These results also indicated that the modification (presumably phosphorylation) of the Ser/Thr site in the activation loop of OsMKK4 is necessary for activation of OsMPK3 and OsMPK6 *in vivo*. OsMPK4 was not activated after DEX induction and was therefore not considered to be a target of OsMKK4 or OsMKK5.

### OsMKK4^DD^ induces cell death but not ROS production

**Figure 3:**
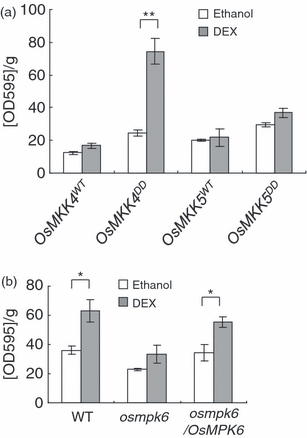
Regulation of cell death by OsMKK4^DD^.Evans blue staining of cells treated with 10 μm dexamethasone (DEX) or ethanol (as a negative control) for 48 h. The asterisks indicate significant differences between staining levels after the same cells were treated with ethanol or with DEX, based on a Student’s *t*-test (*P<0.05, **P<0.01). Two independent cell lines were used for each construct, and three independent experiments were performed, with reproducible results. The bars represent the means of data from a representative experiment, and the error bars indicate standard deviations (*n*=3).In (a) wild-type (WT) cells expressing *OsMKK4*^*WT*^, *OsMKK4*^*DD*^, *OsMKK5*^*WT*^ and *OsMKK5*^*DD*^ were analyzed.In (b) WT, *osmpk6* and *osmpk6*/*OsMPK6* cells expressing *OsMKK4*^*DD*^ were analyzed.

Several MAMPs induce cell death in cultured rice cells (Desaki *et al.*, 2006). We examined whether cell death occurs in cultured cells expressing the DEX-inducible MAPKKs using Evans blue staining. We observed that some of the cells expressing *OsMKK4*^*DD*^ were dead at 48 h after the beginning of DEX treatment (Figure 3a). None of the cells expressing other MAPKK constructs died. Transgenic rice seedlings expressing the same DEX-inducible *OsMKK4*^*DD*^ construct stopped growing and their roots became brown 2 days after transfer to a DEX-containing medium (Figure S8). Seven days after the transfer, whole plants had completely died, while vector control and *OsMKK4*^*WT*^ plants continued to grow.

We generated transgenic *osmpk6* and *osmpk6*/*OsMPK6* cells expressing HA-tagged *OsMKK4*^*DD*^ under the control of the DEX-inducible promoter. All the cell lines accumulated OsMKK4^DD^ protein after the DEX treatment (Figure S9). The *osmpk6*/*OsMPK6* cells expressing *OsMKK4*^*DD*^ accumulated OsMPK6 protein at a level similar to that in WT cells. In the *osmpk6* cells expressing *OsMKK4*^*DD*^, the OsMPK6 kinase activity disappeared but a 43-kDa band, presumably due to OsMPK3, increased after DEX treatment. We examined whether cell death occurs after induction of OsMKK4^DD^ in these cells (Figure 3b). The results showed that WT and *osmpk6*/*OsMPK6* cells, but not *osmpk6* cells, were dead by 48 h after the beginning of OsMKK4^DD^ induction by DEX treatment. These results demonstrate that OsMPK6 is essential for OsMKK4^DD^-induced cell death.

**Figure 4:**
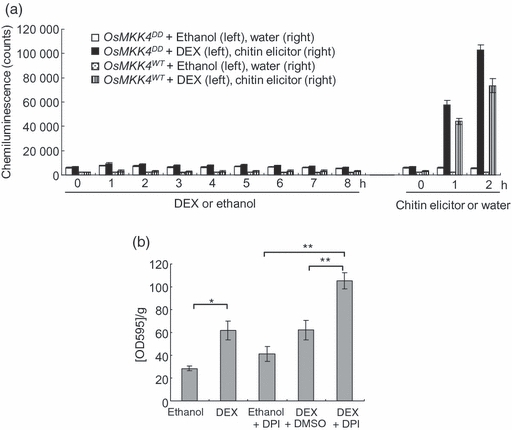
Expression of *OsMKK4*^*DD*^ did not induce production of reactive oxygen species (ROS).(a) Production of ROS was measured in cells harboring dexamethasone (DEX)-inducible *OsMKK4*^*WT*^ and *OsMKK4*^*DD*^ constructs. The cells were treated with DEX, ethanol (control for DEX), chitin elicitor or water (control for elicitor) for the times indicated.(b) Evans blue staining of *OsMKK4*^*DD*^ cells treated with 10 μm DEX and 2 μm diphenyleneiodonium (DPI). DMSO was used as a solvent control for DPI. The asterisks indicate significant differences between staining levels after the same cells were treated with ethanol or with DEX, based on a Student’s *t*-test (*P<0.05, **P<0.01). The bars represent means with standard deviations (*n*=3).

Reactive oxygen species play critical roles in defense responses, including HR cell death during plant–pathogen interactions (Lamb and Dixon, 1997). In tobacco and Arabidopsis, transient expression of active MAPKKs induces ROS production (Ren *et al.*, 2002; Liu *et al.*, 2007). To test whether ROS production was induced by OsMKK4^DD^, we analyzed extracellular ROS levels using a luminol-based chemiluminescence detection system. For a positive control, we used chitin elicitor that is known to induce ROS production in suspension-cultured rice cells (Kuchitsu *et al.*, 1995). Extracellular ROS levels did not increase in cells expressing the DEX-inducible *OsMKK4*^*DD*^ or *OsMKK4*^*WT*^ constructs after treatment with DEX, ethanol or water. However, the same cell lines treated with chitin elicitor showed dramatically enhanced ROS production (Figure 4a). In *Nicotiana benthamiana*, an active MAPKK induces ROS production and the gene for a respiratory burst oxidase homolog (Rboh), which is an NADPH oxidase (Asai *et al.*, 2008). To examine whether Rboh activity is involved in the cell death induced by activation of OsMKK4, we tested the effect of the NADPH oxidase inhibitor diphenyleneiodonium (DPI). However, DPI did not suppress cell death, but rather increased it, in cells expressing *OsMKK4*^*DD*^ (Figure 4b). Therefore, we concluded that the induction of cell death by OsMKK4^DD^ is not associated with extracellular ROS or Rboh activity.

We also examined whether OsMKK4 induces ROS production and HR cell death in *N. benthamiana. OsMKK4*^*DD*^, *OsMKK4*^*WT*^ and *NtMEK2*^*DD*^ were transiently expressed in *N. benthamiana* leaves using the *Agrobacterium tumefaciens* infiltration method. The infiltrated leaves were stained with 3,3′-diaminobenzidine (DAB), which is used to detect ROS. The parts of leaves expressing *OsMKK4*^*DD*^ became brown with DAB staining 2 days after infiltration and were dead at 4 days after infiltration (Figure S10). This result indicates that OsMKK4 has the potential to induce production of ROS and HR cell death in *N. benthamiana*, as its tobacco homolog does.

### Activation of OsMKK4 induced a broad array of changes in gene expression

To identify downstream responses to the activation of OsMKK4, we performed a genome-wide transcript analysis. Gene expression was compared in cells expressing the DEX-inducible *OsMKK4*^*DD*^ construct and cells harboring the control vector. Cells expressing the DEX-inducible *OsMKK4*^*WT*^ construct were also used as a control. We performed statistical analyses of the normalized data (Appendix S1) and chose genes that were up-regulated and down-regulated, respectively, by OsMKK4^DD^ at 12 h after the onset of DEX treatment (Figure S11, Table S1). We categorized these OsMKK4^DD^-regulated genes manually or with gene ontology (GO) classifications (Tables 1 and S2).

**Table 1:** Summary of OsMKK4^DD^-regulated genes

		Percentage of genes classified based on OsMPK6 dependence^a^		
	Number of OsMKK4^DD^-regulated genes in WT cell	I	II	III
Up-regulated genes	2147	28.8	54.6	11.7
Defense related gene	42	31.9	51.1	14.9
Transcription factor	129	39.7	45.8	4.6
Protein kinase	171	31.6	55.9	10.2
Aromatic compound metabolism	54	47.5	42.4	6.8
Sugar metabolism	27	21.4	57.1	21.4
Isoprenoid metabolism	17	25.0	68.8	6.3
Down-regulated genes	2116	6.2	86.6	5.6
Protein biosynthesis	203	0.4	74.9	22.1
Cell cycle	37	0	94.7	5.3

Detailed information can be found in Tables S1 and S2.

a Classes I, II and III; see text for details.

WT, wild type.

The genes that were up-regulated by OsMKK4^DD^ included defense-related genes and genes encoding transcription factors and protein kinases, such as *OsMPK3* and *receptor-like kinase (RLK)* genes. We also found many genes which were annotated by GO with functions in sugar metabolism, aromatic-compound metabolism and isoprenoid metabolism. Genes annotated with functions in sugar metabolism included those for enzymes in the glycolysis pathway leading to pyruvate biosynthesis. Pyruvate and phosphoenolpyruvate are the precursors for terpenoid biosynthesis and the shikimate pathway, respectively. OsMKK4^DD^ increased the transcript levels of these glycolysis pathway genes (Figure S12, Table S3).

Many of the genes down-regulated by OsMKK4^DD^ were annotated with functions in protein biosynthesis, such as tRNA synthetase, initiation factor, elongation factor and ribosomal proteins. More than half of the ribosomal protein genes in the rice genome were down-regulated by OsMKK4^DD^ (Table S4). In addition, genes implicated in the cell cycle, including some encoding histones and cyclins, were down-regulated by OsMKK4^DD^. The coordinated repression of these genes implies that OsMKK4 regulates the suppression of basic cellular activities.

### OsMPK6-dependence of the OsMKK4^DD^-induced gene expression

**Figure 5:**
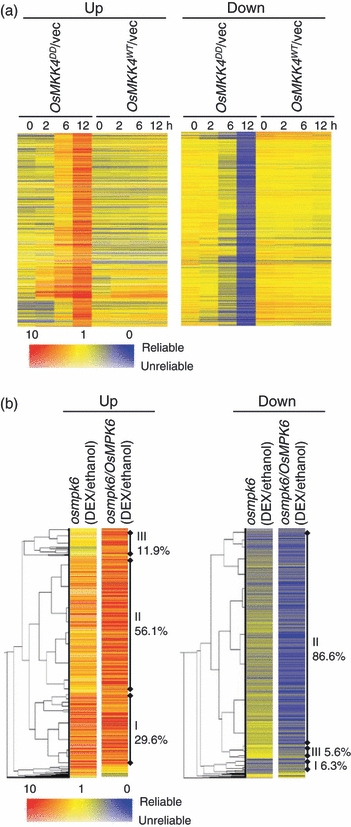
Summary of the genes regulated by OsMKK4^DD^.In the heat maps, colors represent repression (blue) and induction (red) as indicated by the color bar. Relatively more reliable signals and less reliable signals are shown in darker and paler colors, respectively. Data are means of three biological replicates.(a) Heat map of the gene expression ratios for the mitogen-activated protein kinase kinase (MAPKK) constructs versus empty vector over a time course of dexamethasone (DEX) treatment. Each row represents a gene that was up- or down-regulated by OsMKK4^DD^ at 12 h after the beginning of DEX treatment.(b) Hierarchical clustering of gene expression ratios in the *osmpk6* and *osmpk6*/*OsMPK6* backgrounds. Each row indicates the expression ratio of a regulated gene after DEX treatment versus ethanol treatment (DEX/ethanol). Similarities were measured using the Pearson correlation with average linkage as a clustering algorithm. The classes of genes (I, II and III; see text for details) and their percentages are indicated at the right.

We systemically analyzed OsMPK6-dependence of OsMKK4^DD^-induced gene expression. The *osmpk6* and *osmpk6*/*OsMPK6* cells harboring the *OsMKK4*^*DD*^ construct driven by the DEX-inducible promoter were treated with DEX or ethanol as a control. There were only small differences in expression of OsMKK4^DD^-regulated genes in the *osmpk6* and *osmpk6*/*OsMPK6* cells treated with ethanol (Figure S13). Then, we compared OsMKK4^DD^-induced changes in gene expression at 12 h after DEX treatment. We classified the genes that showed OsMKK4^DD^-induced changes in expression in the two different backgrounds as follows: (i) OsMKK4^DD^-induced changes were nearly equal; (ii) OsMKK4^DD^-induced changes in the *osmpk6* background were smaller than those in the *osmpk6*/*OsMPK6* background; (iii) OsMKK4^DD^-induced changes occurred in the *osmpk6*/*OsMPK6* background but not the *osmpk6* background (Figure 5b, Tables 1 and S1). Notably, a much smaller proportion of OsMKK4^DD^-down-regulated genes fell into class I as compared with OsMKK4^DD^-up-regulated genes, indicating that the down-regulated genes are more dependent on OsMPK6 than the up-regulated genes. Classification of the genes in each functional category (Table 1) showed that expression of OsMKK4^DD^-regulated genes that function in isoprenoid metabolism, protein biosynthesis and the cell cycle were more dependent on OsMPK6 than those in other categories.

### OsMKK4^DD^ regulates diterpenoid phytoalexin biosynthesis

**Figure 6:**
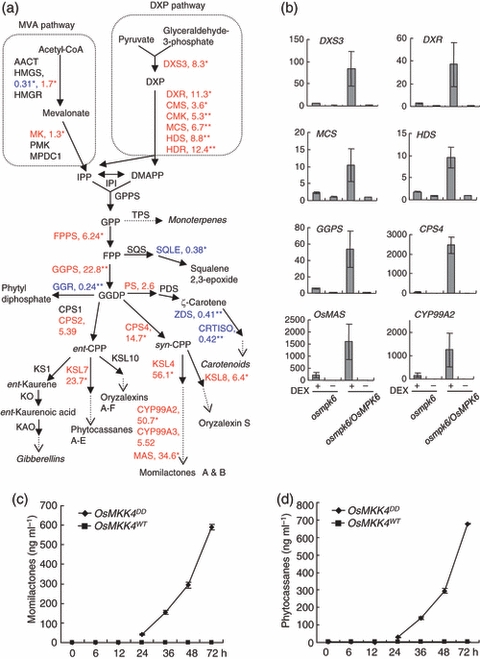
Regulation of diterpenoid phytoalexin biosynthesis by OsMKK4^DD^.(a) The isoprenoid synthesis and diterpenoid metabolism pathways, showing the enzymes whose genes were up- or down-regulated by OsMKK4^DD^ in wild-type (WT) cells. The numbers indicate gene expression ratios (*OsMKK4*^*DD*^ versus vector control) at 12 h after the beginning of dexamethasone (DEX) treatment. Red and blue characters indicate up- and down-regulation, respectively. The asterisks indicate the significance levels according to the one-sample *t*-test (*P<0.05, **P<0.01).(b) Bar graphs of gene expression ratios derived from the microarray data set for *osmpk6* and *osmpk6*/*OsMPK6* cells expressing *OsMKK4*^*DD*^ (+DEX) compared with the same cells treated with ethanol (–DEX) for 12 h.(c), (d) Levels of momilactone A and B (c) and phytocassane A–E (d) in cells after treatment with 10 μm DEX. Two independent cell lines were used for each construct and three independent experiments were performed for each line, with reproducible results. The graphs represent the means of data from a representative experiment, and the error bars indicate standard deviations.Abbreviations: AACT, acetoacetyl-CoA thiolase; CMK, 4-(cytidine 5′-diphospho)-2-*C*-methyl-d-erythritol kinase; CMS, 4-(cytidine 5′-diphospho)-2-*C*-methyl-d-erythritol synthase; CPP, copalyl diphosphate; CPS, copalyl diphosphate synthase; CRTISO, carotenoid isomerase; DMAPP, dimethylallyl diphosphate; DXP, 1-deoxy-d-xylulose 5-phosphate; DXR, DXP reductoisomerase; DXS, DXP synthase; FPP, farnesyl diphosphate; FPPS, FPP synthase; GGDP, geranylgeranyl diphosphate; GGR, geranylgeranyl reductase; GGPS, geranylgeranyl diphosphate synthase; GPP, geranyl diphosphate; GPPS, geranyl diphosphate synthase; HDS, HMBDP synthase; HDR, HMBDP reductase; HMBDP, 1-hydroxy-2-methyl-2-(*E*)-butenyl4-diphosphate; HMGR, 3-hydroxy-3-methylglutaryl-CoA reductase; HMGS, 3-hydroxy-3-methylglutaryl-CoA synthase; IPI, isopentenyl diphosphate isomerase; IPP, isopentenyl diphosphate; KAO, *ent*-kaurenoic acid oxidase; KO, *ent*-kaurene oxidase; KS, kaurene synthase; MAS, momilactone A synthase; MCS, 2-*C*-methyl-d-erythritol 2,4-cyclodiphosphate synthase; MK, mevalonate kinase; MPDC, mevalonate diphosphate decarboxylase; PDS, phytoene desaturase; PMK, phosphomevalonate kinase; PS, phytoene synthase; SQLE, squalene epoxidase; SQS, squalene synthase; TPS, monoterpene/sesquiterpene synthase-like genes; ZDS, zeta-carotene desaturase.

The genes involved in isoprenoid biosynthesis were found to be up-regulated by OsMKK4^DD^ in WT cells. In higher plants, two distinct pathways, the mevalonate (MVA) pathway and the DXP pathway, lead to isoprenoid biosynthesis (Lichtenthaler *et al.*, 1997). Chitin elicitor induces the activation of DXP pathway genes leading to diterpenoid phytoalexin biosynthesis but not MVA pathway genes (Okada *et al.*, 2007). Diterpenoid pathway genes have been extensively characterized in rice (Sakamoto *et al.*, 2004; Okada *et al.*, 2007; Shimura *et al.*, 2007). We searched for other genes in the isoprenoid metabolism pathways using databases, and examined their expression levels (Figure 6a, Table S5). While the genes in the MVA pathway were not significantly changed, most of the genes encoding enzymes in the DXP pathway were up-regulated. GGDP is positioned at the branching point of the GA and phytoalexin biosynthesis pathways, and is sequentially cyclized by CPS and KS or KS-like (KSL) enzymes to yield diterpene hydrocarbons. The *CPS2*, *CPS4*, *KSL4*, *KSL7* and *KSL8* genes, which are involved in phytoalexin synthesis (Peters, 2006), were up-regulated by OsMKK4^DD^, while the *CPS1* and *KS1* genes, which are involved in GA synthesis, were not. These results indicate that OsMKK4^DD^ specifically induces the genes in the diterpenoid phytoalexin synthetic pathway. *OsTGAP1* is up-regulated by the chitin elicitor and induces the expression of genes involved in momilactone biosynthesis (Okada *et al.*, 2009). However, we did not find any increase in *OsTGAP1* transcripts after OsMKK4^DD^ induction (Table S5).

We also compared the expression patterns of DXP pathway and diterpenoid pathway genes with or without OsMKK4^DD^ in the *osmpk6* and *osmpk6*/*OsMPK6* backgrounds (Figure 6b, Table S5). These genes showed only small OsMKK4^DD^-dependent changes of expression in the *osmpk6* background. Thus, OsMPK6 plays the major role in the OsMKK4^DD^-induced expression of diterpenoid pathway genes.

Since the gene expression profiles indicated that diterpenoid phytoalexin biosynthesis is specifically regulated by activated OsMKK4, we analyzed the accumulation of momilactones and phytocassanes in cells expressing *OsMKK4*^*DD*^ and *OsMKK4*^*WT*^. Momilactones and phytocassanes began to accumulate at 24 h after the beginning of DEX treatment and continued to increase in the cells expressing *OsMKK4*^*DD*^ (Figure 6c,d). The cells expressing *OsMKK4*^*WT*^ did not accumulate these phytoalexins. Taken together, we conclude that activated OsMKK4 coordinately induces multiple genes in the DXP and diterpenoid phytoalexin pathways, thereby driving metabolic flow to diterpenoid phytoalexin biosynthesis.

### OsMKK4^DD^ regulates phenylpropanoid biosynthesis and lignin accumulation

In many plants, toxic metabolites are produced from tryptophan (Trp) or phenylalanine (Phe). Phenylalanine is the carbon source for phenylpropanoid biosynthesis and is converted by phenylalanine ammonia-lyase (PAL) to cinnamic acid, which is further modified to *p*-coumaroyl-CoA. Most phenylpropanoid compounds such as lignin, flavonoids and many small phenolic molecules are derived from *p*-coumaroyl-CoA. In rice, some Trp-derived metabolites (Ishihara *et al.*, 2008) and lignin (Cai *et al.*, 2008) increase after pathogen infection.

**Figure 7:**
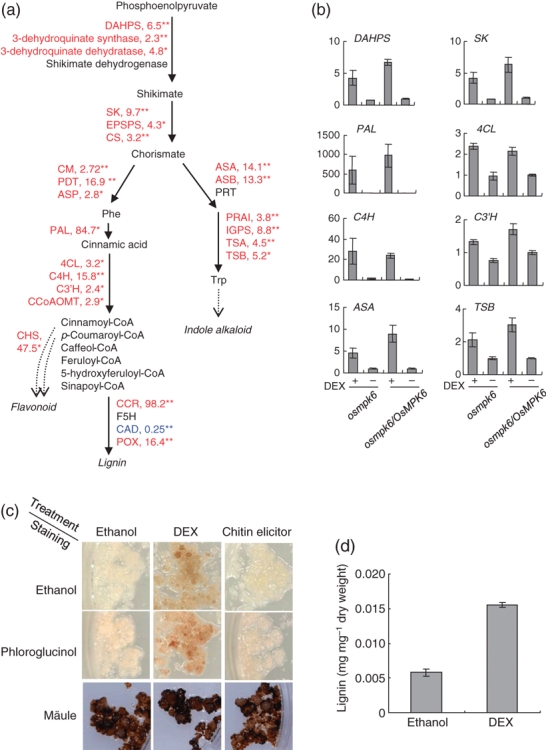
Regulation of phenylpropanoid biosynthesis by OsMKK4^DD^.(a) The shikimate pathway, and pathways for aromatic amino acid metabolism and phenylpropanoid biosynthesis, showing enzymes whose genes were up- or down-regulated by OsMKK4^DD^ in wild-type (WT) cells. The numbers indicate gene expression ratios (*OsMKK4*^*DD*^ versus vector control) at 12 h after beginning of dexamethasone (DEX) treatment. Red and blue characters indicate up- and down-regulation, respectively. The asterisks indicate the significance levels according to the one-sample *t*-test (*P<0.05, **P<0.01).(b) Bar graphs of gene expression ratios derived from the microarray data set for *osmpk6* and *osmpk6*/*OsMPK6* cells expressing *OsMKK4*^*DD*^ (+DEX) compared with the same cells treated with ethanol (–DEX) for 12 h.(c) Lignin staining of cells containing the DEX-inducible *OsMKK4*^*DD*^ construct treated for 3 days with 10 μm DEX, ethanol (control for DEX) or chitin elicitor. Cells expressing *OsMKK4*^*DD*^ accumulated brown material even without phloroglucinol treatment, and turned red after staining with phloroglucinol.(d) Lignin levels in cells expressing *OsMKK4*^*DD*^ at 3 days after DEX treatment. The bars represent the means of data from a representative experiment, and the error bars indicate standard deviations (*n*=3). For (c) and (d), two independent cell lines were used and three independent experiments were performed with reproducible results.Abbreviations: 4CL, 4-coumarate-CoA ligase; ADH, arogenate dehydrogenase; ASA, anthranilate synthase α subunit; ASB, anthranilate synthase β subunit; ASP, aspartate aminotransferase; CAD, cinnamyl-alcohol dehydrogenase; CCoAOMT, caffeoyl-CoA *O*-methyltransferase; CCR, cinnamoyl-CoA reductase; CHS, chalcone synthase; CM, chorismate mutase; CS, chorismate synthese; C3′H, *p*-coumarate 3-hydroxylase, *p*-coumaroyl shikimate/quinate 3′-hydroxylase; C4H, *trans*-cinnamate 4-hydroxylase; DAHPS, 3-deoxy-d-*arabino*-heptulosonate 7-phosphate synthase; EPSPS, 5-enolpyruvylshikimate 3-phosphate synthase; F5H, ferulate 5-hydroxylase; IGPS, indole-3-glycerol phosphate synthase; PAL, phenylalanine ammonia-lyase; PDT, prephenate dehydratase; PRAI, phosphoribosylanthranilate isomerase; PRT, phosphoribosylanthranilate transferase; POX, peroxidase; SK, shikimate kinase; TSA, tryptophan synthase α subunit; TSB, tryptophan synthase β subunit.

A GO analysis of the OsMKK4^DD^-up-regulated genes revealed that the genes encoding enzymes involved in the metabolism of aromatic compounds, which includes Trp and Phe, were up-regulated by OsMKK4^DD^. Therefore, we searched for other genes whose products are predicted to function in these pathways, and found that several genes were up-regulated (Figure 7a, Table S6). Although one of *CAD* genes was down-regulated by OsMKK4^DD^, other *CAD* genes were unaffected (Table S6).

We also compared the expression of these genes with or without OsMKK4^DD^ induction in the *osmpk6* and *osmpk6*/*OsMPK6* backgrounds (Figure 7b, Table S6). The OsMKK4^DD^-induced changes in the *osmpk6* background were comparable with those in the *osmpk6*/*OsMPK6* background. These results indicate that OsMPK6 is not involved in the OsMKK4^DD^-induced expression of the Trp, Phe and phenylpropanoid pathway genes, unlike those for diterpenoid phytoalexin pathway genes.

The cells expressing *OsMKK4^DD^* became brown 3 days after DEX treatment (Figure 7c). This observation suggests that phenolic materials accumulate after OsMKK4^DD^ induction. Therefore we analyzed lignin accumulation in cells expressing *OsMKK4^DD^* and in the same cells treated only with chitin elicitor, using phloroglucinol and Mäule staining (Figure 7c). The cells expressing *OsMKK4*^*DD*^ were stained red by phloroglucinol, suggesting that these cells accumulated hydroxycinnamaldehydes, precursors of monolignols. When the Mäule stain was used, all the cells were stained black and there were no differences between the treatments. We also measured the amounts of thioglycolic acid (TGA)-extractable cell-wall complexes that are commonly thought to be lignin (Kawasaki *et al.*, 2006). The amount of TGA-extractable lignin was higher in the cells expressing *OsMKK4*^*DD*^ than in ethanol-treated cells (Figure 7d). These results indicate that activated OsMKK4 induces lignin accumulation. The *osmpk6* cells expressing OsMKK4^DD^ also became brown 3 days after DEX treatment (Figure S14), which suggests that phenolic materials also accumulate in these cells, consistent with the results of transcript profiling.

Genes encoding chalcone synthase were highly up-regulated in cells expressing *OsMKK4^DD^*, implying the accumulation of flavonoids. However, we detected no change in the level of the flavonoid-type phytoalexin sakuranetin in cells expressing *OsMKK4^DD^* (data not shown).

## Discussion

### OsMKK4–OsMPK3/OsMPK6 cascades mediate the MAMP signal to regulate defense responses

In this work we have shown that OsMPK3, OsMPK4, OsMPK6 and OsMKK4 are activated by the MAMP signal in rice. OsMKK4 activates OsMPK3 and OsMPK6, thereby forming a signaling cascade that induces immune responses such as defense-related gene expression, synthesis of antimicrobial compounds and cell death, but not ROS production. OsMPK6 was found to play a major role in the chitin-elicitor-triggered and OsMKK4-mediated regulation of diterpenoid phytoalexin synthesis and plant cell death, but not that of phenylpropanoid synthesis.

OsMKK4^DD^ induced cell death without extracellular ROS in rice cells, while OsMKK4^DD^ induced cell death and ROS production in *N. benthamiana*. In tobacco and Arabidopsis, active MAPKK induces cell death and ROS production (Yang *et al.*, 2001; Ren *et al.*, 2002). Thus, MAMP-triggered ROS production seems to be regulated differently between rice and tobacco/Arabidopsis. We also showed that the Rboh inhibitor DPI did not inhibit the cell death induced by OsMKK4^DD^. This is consistent with the observation that the suppression of production of extracellular ROS by DPI does not prevent MAMP-triggered cell death in tobacco (Ichinose *et al.*, 2001). It is also known that Rboh inhibits salicylic acid-dependent pro-death signals in Arabidopsis (Torres *et al.*, 2005). Taken together, OsMKK4^DD^-induced cell death in rice is not the result of the generation of extracellular ROS.

The gene for a transcription factor *OsNAC4*, which induces cell death in rice protoplasts (Kaneda *et al.*, 2009), was regulated by OsMKK4^DD^ (Table S1a), raising the possibility that OsMKK4^DD^-induced cell death was mediated by OsNAC4. However, OsMKK4^DD^-induced expression of *OsNAC4* was independent of OsMPK6, whereas OsMKK4^DD^-induced cell death was dependent on OsMPK6. It could be that phosphorylation of OsNAC4 by OsMPK6 is required for OsMKK4^DD^-induced cell death but OsMKK4^DD^-induced transcriptional up-regulation of *OsNAC4* is independent of OsMPK6. We did not find any other cell death-related genes that could account for the difference in the cell death phenotype after OsMKK4^DD^ induction between the *osmpk6*/*OsMPK6* and *osmpk6* backgrounds. Rather, it seems possible that the OsMPK6-dependent down-regulation of genes involved in basic cellular activities, such as tRNA synthetase, initiation factor, elongation factor, ribosomal protein, histone protein and cyclin, is responsible for the OsMKK4^DD^-induced cell death in WT and *osmpk6*/*OsMPK6* cells.

OsMKK4^DD^ also induced *OsMPK3* and numerous genes for receptor-like kinases (RLKs). NtMEK2^DD^ induces *WIPK* expression in tobacco (Liu *et al.*, 2003). Many plant species respond to pathogen infection or treatment with MAMPs with the induction of *OsMPK3* or homologs of *OsMPK3* (Romeis *et al.*, 1999; Xiong *et al.*, 2001) and *RLK* genes (Yoshimura *et al.*, 1998; Zipfel *et al.*, 2004, 2006). In addition, several types of RLK have been identified as MAMP receptors (Gomez-Gomez and Boller, 2000; Miya *et al.*, 2007; Kanzaki *et al.*, 2008). Taken together, these results suggest that OsMKK4 mediates the MAMP-triggered induction of *OsMPK3* and *RLK* genes, thereby preparing plants for the perception of other MAMP signals.

### The OsMKK4–OsMPK3/OsMPK6 cascade regulates metabolic pathways leading to the synthesis of antimicrobial compounds at the transcriptional level

The OsMKK4–OsMPK3/OsMPK6 cascade up-regulates the genes involved in sugar metabolism pathways. Generally, pathogen infection changes carbohydrate metabolism and photosynthesis (Ehness *et al.*, 1997; Berger *et al.*, 2004), with incompatible pathogens inducing these responses more strongly and rapidly (Swarbrick *et al.*, 2006). A deficiency of cell-wall invertase results in the impairment of *PR* gene expression, PAL activity and hypersensitive cell death in tobacco (Essmann *et al.*, 2008). Taken together, the OsMKK4-regulated up-regulation of sugar metabolism genes leads to immediate changes in sugar catabolism, which are required for the biosynthesis of antimicrobial compounds during the immune response.

During evolution, plant–microbe interactions have been a driving force for plants to widen the variety of secondary metabolites that they produce. For example, Arabidopsis, rice, corn, oat and soybean are rich sources of antimicrobial indoles, diterpenoids, benzoxazinones, triterpenenoids and flavonoids/isoflavonoids, respectively. In Arabidopsis, synthesis of an indole-derived phytoalexin, camalexin, is regulated by the AtMPK3/AtMPK6 cascade (Ren *et al.*, 2008). We showed that chitin-elicitor-induced synthesis of diterpenoid phytoalexins is regulated by the OsMKK4–OsMPK6 cascade. While molecular types of phytoalexin are different, induction of phytoalexin biosynthesis seems to be a common function in rice and Arabidopsis MAPK. Analysis of the regulatory mechanism of phytoalexin biosynthesis genes by MAPK will clarify how each plant acquires the ability to induce its specific antimicrobials.

*OsTGAP1* is an elicitor-responsive transcription factor and induces the expression of momilactone biosynthesis genes (Okada *et al.*, 2009), but its transcripts were not induced by OsMKK4^DD^. A number of transcription factors have been reported to be phosphorylated by MAPKs *in vitro* (Feilner *et al.*, 2005; Menke *et al.*, 2005; Yap *et al.*, 2005; Popescu *et al.*, 2009) and *in vivo* (Lampard *et al.*, 2008; Yoo *et al.*, 2008). Possible regulation of transcription factors such as OsTGAP1 via phosophorylation accounts for the accumulation of momilactone and its biosynthesis genes by OsMKK4^DD^ and seems to be one of the important functions of the OsMKK4–OsMPK6 cascade.

Phenylpropanoid derivatives are also widely used for defense in plants. MAMP-triggered increases in lignin have been observed in various plant species, such as pine, spruce and flax (Campbell and Ellis, 1992; Lange *et al.*, 1995; Hano *et al.*, 2006), and this is considered to be a conserved defense response in plants. The MAPK cascade-dependent regulation of *PAL*, a gene for an early step in lignin biosynthesis, has been reported in tobacco (Yang *et al.*, 2001). We showed in rice that OsMKK4^DD^ induced coordinated up-regulation of phenylpropanoid pathway genes leading to lignin accumulation. Thus, the induction of phenylpropanoid pathway genes is a conserved function of MAMP-responsive MAPK cascades in plants.

### Possible redundancy of the OsMKK4–OsMPK3/OsMPK6 cascade

The comparison of OsMKK4^DD^-induced gene expression in the *osmpk6* and *osmpk6*/*OsMPK6* backgrounds revealed a regulatory pathway mediated by the OsMKK4–OsMPK6 cascade. The expression of the genes involved in diterpenoid phytoalexin biosynthesis, protein biosynthesis and the cell cycle were largely dependent of OsMPK6. By contrast, phenylpropanoid biosynthesis genes were regulated in an OsMPK6-independent manner. These results suggest that other MAPK cascade components, such as OsMPK3, are redundant with OsMPK6. To address this question, we tried to knockdown *OsMPK3* in *osmpk6* cells expressing *OsMKK4*^*DD*^; however, we were unable to obtain such transformed cells, suggesting that this genotype is lethal. In Arabidopsis, the *atmpk3*/*atmpk6* double mutant shows an embryonic lethal phenotype (Wang *et al.*, 2007), consistent with this notion.

## Experimental procedures

### Plant material, rice transformation, antibodies, protein extraction, protein gel blot analysis, plasmid construction and kinase assays

A detailed description of these experimental procedures can be found in the Appendix S1.

### ROS assays

Approximately 200 mg of transgenic rice cells were transferred aseptically to 5 ml of fresh medium in Petri dishes and incubated overnight at 25°C with shaking at 0.14 ***g***. The next day, the following were added to separate plates for each cell line: 5 μl of 10 mm DEX solution, 5 μl ethanol (control for DEX), chitin elicitor (*N*-acetylchitooctaose) to a final concentration of 10 nm, or sterile water (control for elicitor). The dishes were incubated as described above for various time periods. The experiment was carried out in triplicate. The ROS generated in the reaction mixture were measured using the luminal-dependent chemiluminescence assay (Schwacke and Hager, 1992).

### Cell death assays

Cell death was assayed by Evans blue staining as described previously (Kurusu *et al.*, 2005). Briefly, the cells were incubated in 20-mm well microplates (0.1 g cells and 1.5 ml fresh medium per well) with 0.05% Evans blue (Sigma, http://www.sigmaaldrich.com/) for 10 min and then washed three time with fresh medium over a period of 3 h to remove any unabsorbed dye. Dye that had been absorbed by dead cells was extracted with 50% methanol plus 1% SDS for 1 h at 60°C, and quantified by absorbance at 595 nm.

### RNA isolation, RT-PCR, microarray analysis and data analysis

A detailed description of these materials and methods can be found in Appendix S1. Analyses were performed using RNA prepared from three independent experiments. An Agilent 44K oligoarray (http://www.home.agilent.com/) was used for microarray analysis. OsMKK4^DD^-dependent gene expression was analyzed by the two-color method and OsMPK6-dependent gene expression by the one-color method. The microarray data are deposited under accession number GSE15613 and GSE18787 in the Gene Expression Omnibus database (https://www.ncbi.nlm.nih.gov/projects/geo/).

### Phytoalexin measurements

Media samples (0.5 ml) from rice cell cultures that had been treated with chitin elicitor or DEX were extracted three times with ethyl acetate (0.5 ml each extraction). The combined ethyl acetate extracts were evaporated to dryness. The residues from each sample were dissolved in 1 ml of 79% ethanol, 7% acetonitrile and 0.01% acetic acid. Aliquots (5 μl) of the resulting solutions were subjected to HPLC–ESI-MS/MS as described previously (Shimizu *et al.*, 2008).

### Lignin measurements and histochemical analysis

Rice suspension cells were harvested, freeze-dried and ground in a ball mill (TissueLyser; Qiagen, http://www.qiagen.com/) for 30 sec at 26 Hz with 20 mm stainless steel balls. The lignin content in the resulting powder (20 mg samples) was measured as thioglycolic acid lignin as described previously (Suzuki *et al.*, 2009).

Histochemical analysis of the accumulated hydroxycinnamaldehydes was performed using phloroglucinol staining. Cells were incubated for 3 min in a phloroglucinol solution (2% in 95% ethanol) or 95% ethanol (staining control), treated with 10% HCl for 3 min, and directly photographed using a digital color camera. Histochemical analysis of the lignin monomer was performed using Maüle staining. Cells were treated with 0.5% KMnO_4_ for 10 min, rinsed with water, treated with 10% HCl for 2 min, rinsed with water again, then mounted in concentrated NH_3_.H_2_O and examined immediately.

### Accession numbers

Sequence data from this article can be found in the GenBank/EMBL data libraries under the following accession numbers: AK067339 (OsMPK3), AK111579 (OsMPK4), AK111691 (OsMPK6), AK120525 (OsMKK4), AK099769 (OsMKK5).

## SUPPORTING INFORMATION

**Figure S1.** MBP kinase activities in elicitor-treated and mock-treated WT cells.

**Figure S2.** Immuno-blots using MAPK specific antibodies.

**Figure S3.** MBP kinase activities of OsMPK3, OsMPK4, and OsMPK6 in elicitor-treated and mock-treated WT cells

**Figure S4.** Position of the *Tos17* insertion in the *OsMPK6* locus and *OsMPK6* expression in *osmpk6* cells.

**Figure S5.** Accumulation of OsMPK6 protein in *osmpk6/OsMPK6* cells used for the phytoalexin measurement.

**Figure S6.** OsMKK4 and OsMKK5 are similar to AtMKK4, AtMKK5 and NtMEK2 and activate OsMPK3, OsMPK4 and OsMPK6 *in vitro*.

**Figure S7.** *In vivo* activation of OsMPK3 and OsMPK6 by constitutively active OsMKK4.

**Figure S8.** Phenotypes of seedlings expressing *OsMKK4^DD^* or *OsMKK4^WT^*.

**Figure S9.** Activation of kinases by the expression of OsM*KK4^DD^* in WT*, osmpk6,* and *osmpk6/OsMPK6* cells.

**Figure S10.** Reactive oxygen species ROS and cell death assays in *Nicotiana benthamiana*.

**Figure S11.** Summary of the genes regulated by OsMKK4^DD^.

**Figure S12.** Regulation of sugar metabolism pathway genes by Os MKK4^DD^.

**Figure S13.** Gene regulation in *osmpk6* cells expressing *OsMKK4^DD^*.

**Figure S14.** Appearance of cells after OsMKK4^DD^ induction.

**Table S1.** List of genes significantly regulated by OsMKK4^DD^.

**Table S2.** Classification using Gene Ontology.

**Table S3.** List of genes related to sugar metabolism.

**Table S4.** Ribosomal protein genes.

**Table S5.** List of genes related to isoprenoid metabolism.

**Table S6.** List of genes related to phenylpropanoid metabolism.

**Appendix S1.** Full legends for supporting figures, experimental procedures and references.

Please note: As a service to our authors and readers, this journal provides supporting information supplied by the authors. Such materials are peer-reviewed and may be re-organized for online delivery, but are not copy-edited or typeset. Technical support issues arising from supporting information (other than missing files) should be addressed to the authors.
