# Supplementary material for: A rice fungal MAMP-responsive MAPK cascade regulates metabolic flow to antimicrobial metabolite synthesis
Source: Plant J. 2010 Jun 21;63(4):599–612. doi: 10.1111/j.1365-313X.2010.04264.x (PMC2988419; doi:10.1111/j.1365-313X.2010.04264.x)
Supplement: Supplementary file 1 [file tpj0063-0599-SD1.pdf]

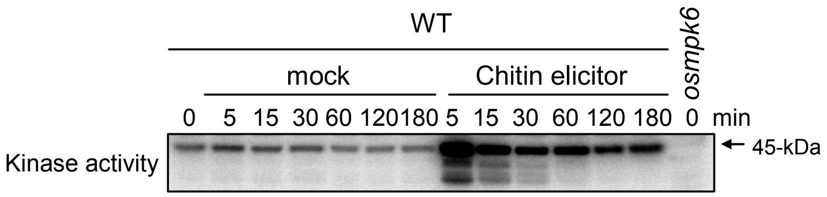

Figure S1 MBP kinase activities in elicitor-treated and mock-treated WT cells.

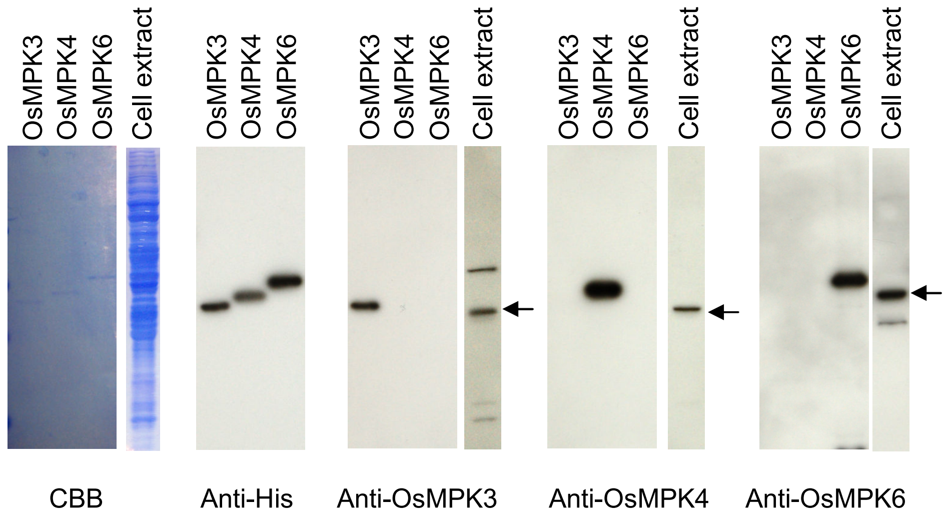

Figure S2 Immuno-blots using MAPK specific antibodies.

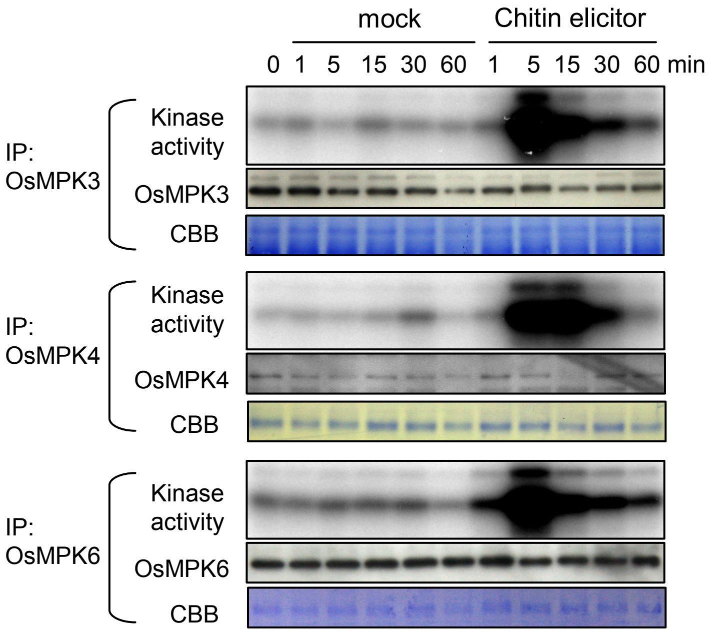

Figure S3 MBP kinase activities of OsMPK3, OsMPK4, and OsMPK6 in elicitor-treated and mock-treated WT cells.

A

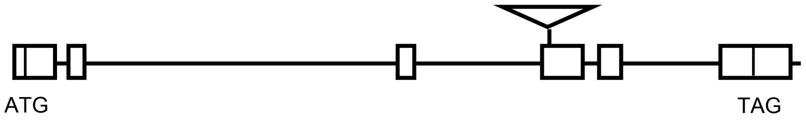

B

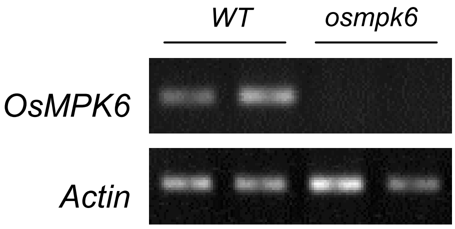

Figure S4 Position of the *Tos17* insertion in the *OsMPK6* locus and *OsMPK6* expression in *osmpk6* cells.

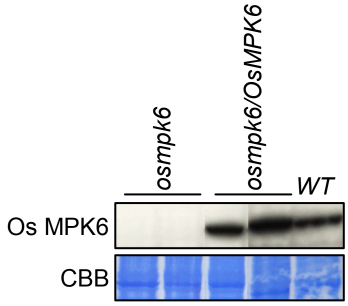

Figure S5 Accumulation of OsMPK6 protein in *osmpk6/OsMPK6* lines used for the phytoalexin measurement.

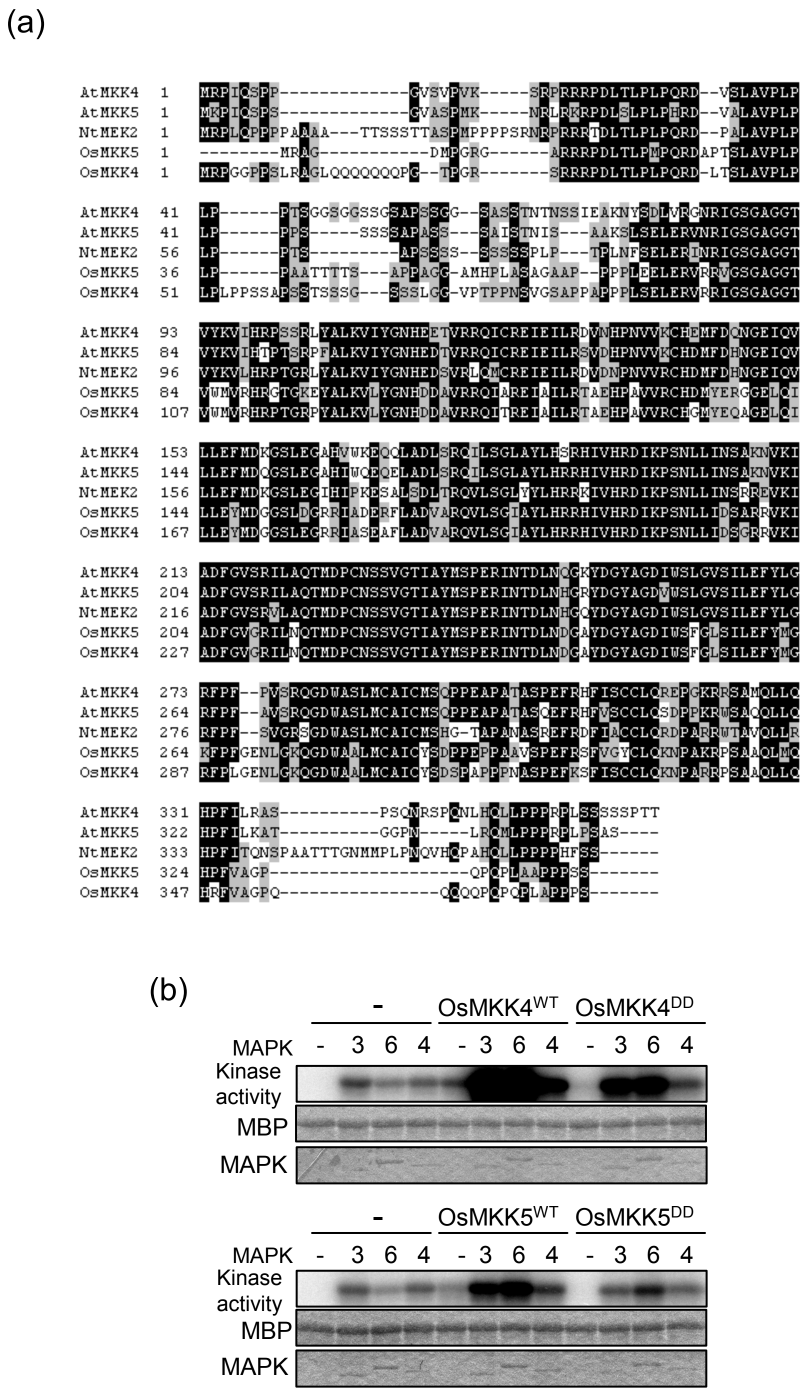

Figure S6 OsMKK4 and OsMKK5 are similar to AtMKK4, AtMKK5 and NtMEK2 and activate OsMPK3, OsMPK4 and OsMPK6 *in vitro*.

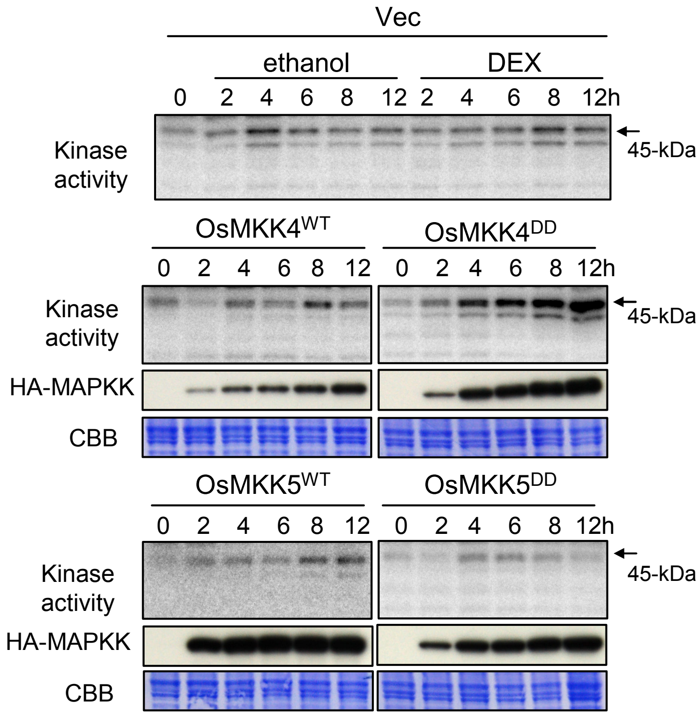

Figure S7 *In vivo* activation of OsMPK3 and OsMPK6 by constitutively active OsMKK4.

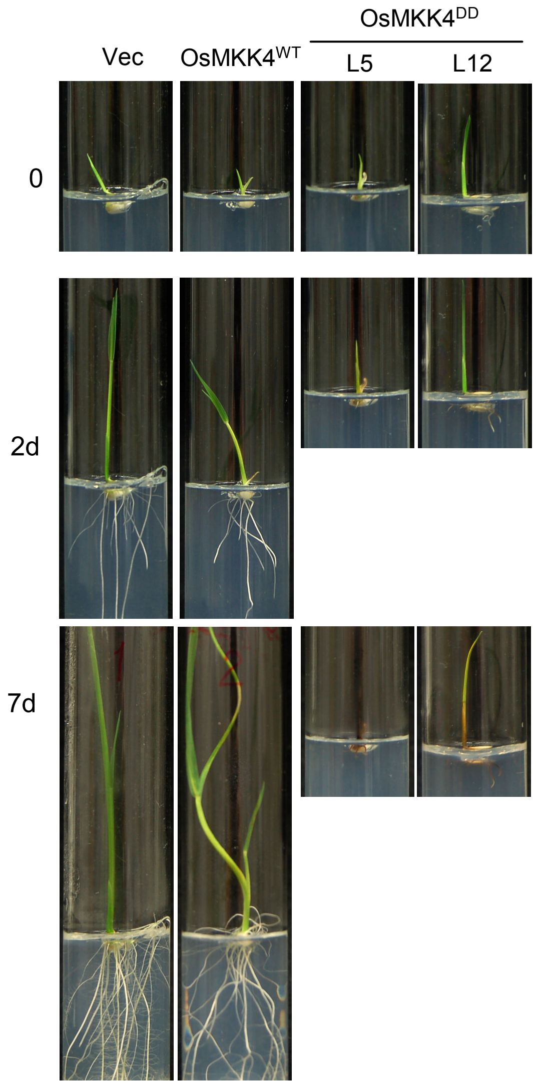

Figure S8 Phenotypes of seedlings expressing *OsMKK4*^{DD} or *OsMKK4*^{WT}.

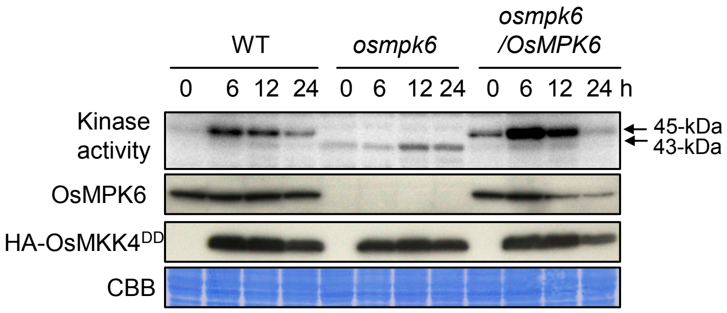

Figure S9 Activation of kinases by the expression of OsM*KK4*^{DD} in WT*,* *osmpk6,* and *osmpk6/OsMPK6* cells.

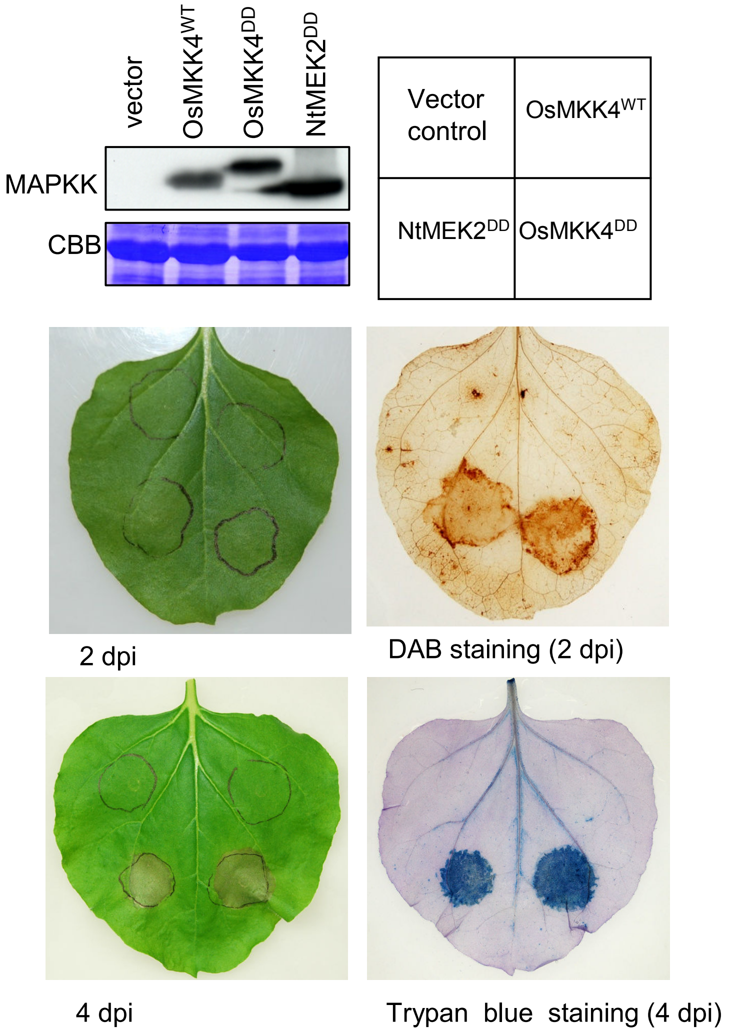

Figure S10 ROS and cell death assays in *Nicotiana benthamiana*.

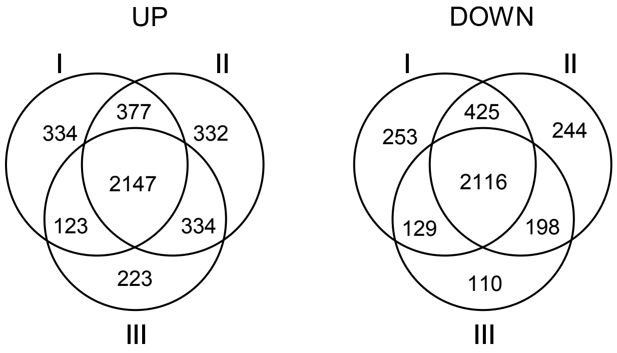

Figure S11 Summary of the genes regulated by OsMKK4^{DD}.

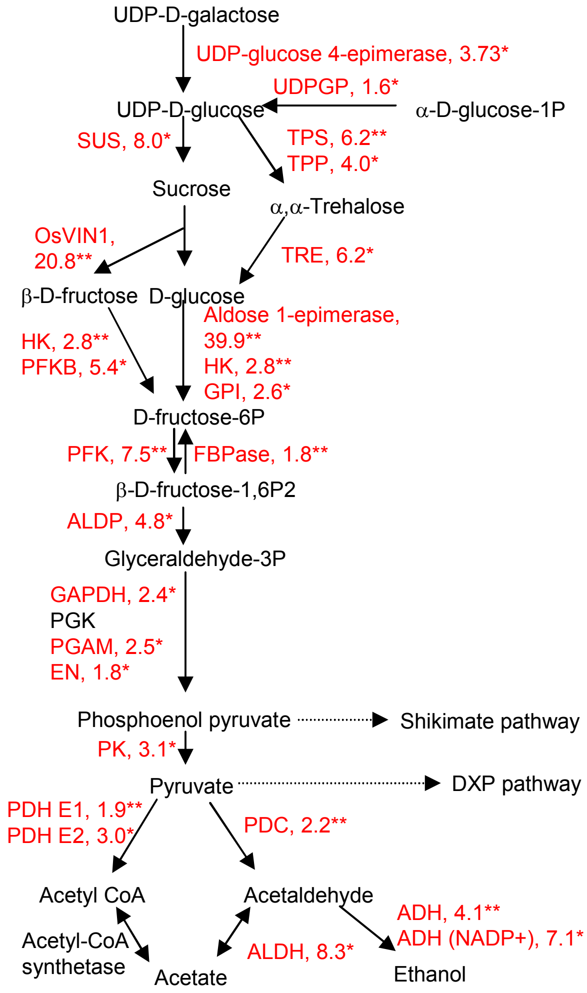

Figure S12 Regulation of sugar metabolism pathway genes by OsMKK4^{DD}.

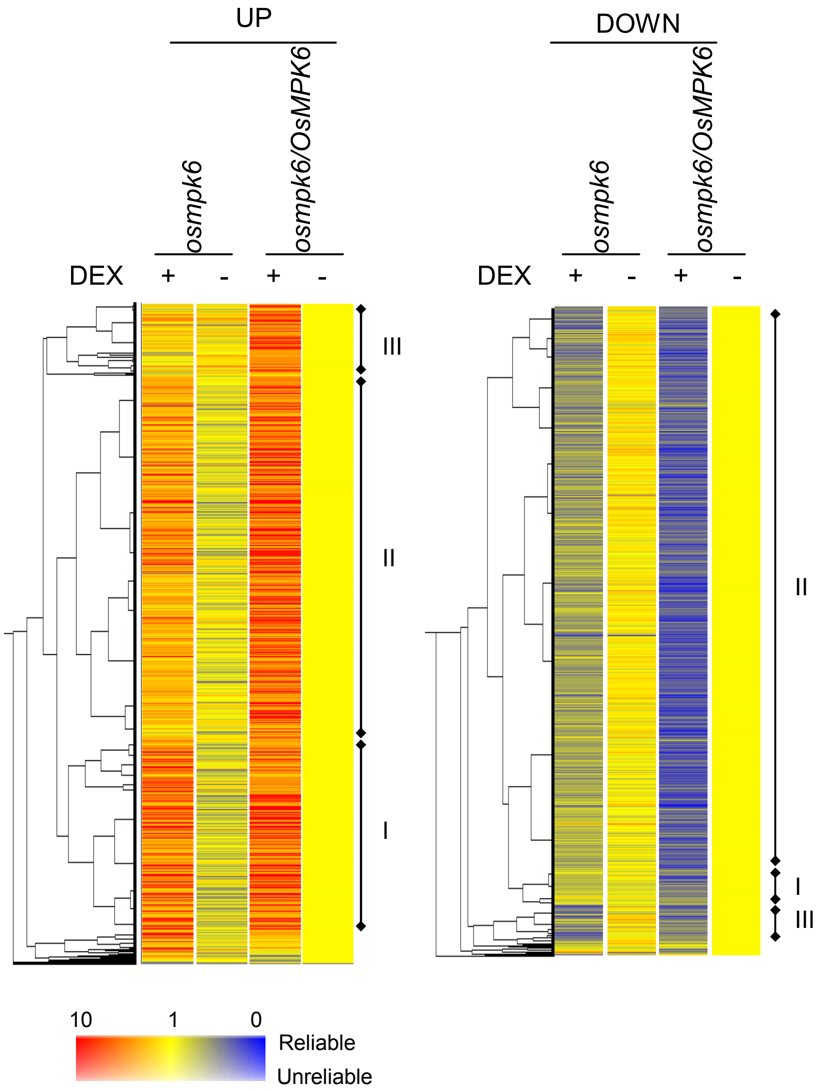

Figure S13 Gene regulation in *osmpk6* cells expressing *OsMKK4*^{DD}.

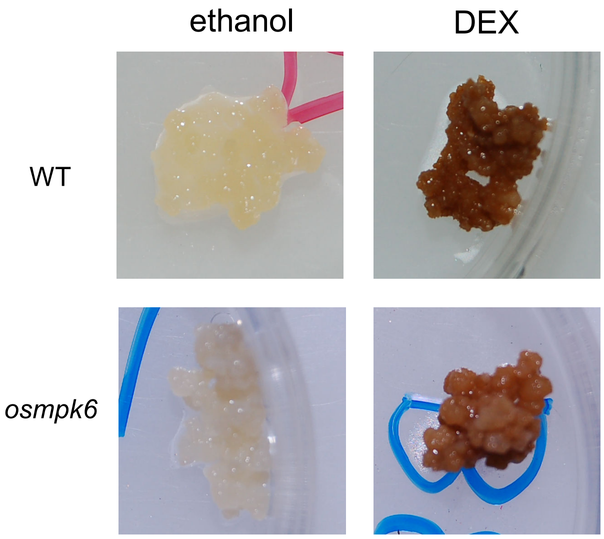

Figure S14 Appearance of cells after OsMKK4^{DD} induction.
