# Supplementary material for: A rice fungal MAMP-responsive MAPK cascade regulates metabolic flow to antimicrobial metabolite synthesis
Source: Plant J. 2010 Jun 21;63(4):599–612. doi: 10.1111/j.1365-313X.2010.04264.x (PMC2988419; doi:10.1111/j.1365-313X.2010.04264.x)
Supplement: Supplementary file 8 [file tpj0063-0599-SD8.doc]

**Legends for supporting figures**

**Figure S1.** MBP kinase activities in elicitor-treated and mock-treated WT cells.

Kinase activities were analyzed using in-gel kinase assays with myelin basic protein (MBP) as a substrate. The arrow indicates the position of OsMPK6 with the size of 45-kDa.

**Figure S2.** Immuno-blots using MAPK specific antibodies.

Specific antibodies against OsMPK3, OsMPK4 and OsMPK6 did not cross react with other MAPKs. Recombinant proteins with N-terminal His-tag and cell extracts were separated by SDS-PAGE, blotted onto membranes and the MAPKs were detected using the specific antibodies.

**Figure S3.** MBP kinase activities of OsMPK3, OsMPK4, and OsMPK6 in elicitor-treated and mock-treated WT cells.

Kinase activation was determined using an IP kinase assay. The kinases were immunoprecipitated from 10 μg (OsMPK6), 50 μg (OsMPK3), or 100 μg (OsMPK4) total proteins using specific antibodies. Phosphorylation of MBP was detected by autoradiography after SDS-PAGE (each top panel). MAPKs were detected by immuno-blot analysis (each middle panel). Equal loading was confirmed by CBB staining (each bottom panel).

**Figure S4.** Position of the *Tos17* insertion in the *OsMPK6* locus and *OsMPK6* expression in *osmpk6* cells.

(a) *Tos17* knockout mutants of *OsMPK6*. Exons are indicated by white boxes. *Tos17* is inserted in the fourth exon of *OsMPK6* locus.

(b) Expression of *OsMPK6* in WT and *osmpk6* mutant cell. *OsMPK6* and control *actin* were analyzed by RT-PCR.

**Figure S5.** Accumulation of OsMPK6 protein in *osmpk6/OsMPK6* cells used for the phytoalexin measurement.

*osmpk6* mutant was transformed with a cDNA of OsMPK6 fused with its promoter sequence. OsMPK6 protein was detected by immuno-blot analysis (top panel). Equal loading was confirmed by CBB staining (bottom panel).

**Figure S6.** OsMKK4 and OsMKK5 are similar to AtMKK4, AtMKK5 and NtMEK2 and activate OsMPK3, OsMPK4 and OsMPK6 *in vitro*.

(a) Protein sequences of AtMKK4, AtMKK5, NtMEK2, OsMKK4 and OsMKK5 were aligned using the ClustalX software.

(b) MBP kinase activities of recombinant OsMPK3, OsMPK4, and OsMPK6, after activation by recombinant OsMKK4 and OsMKK5 *in vitro*. The recombinant OsMPK3, OsMPK4, and OsMPK6 were first activated by OsMKK4 and OsMKK5, and then the active MAPKs were used to phosphorylate MBP *in vitro*. Phosphorylation of MBP was detected by autoradiography after SDS-PAGE (top panel). Equal loading of MBP (middle panel) and MAPKs (bottom panel) was confirmed by CBB staining. Reactions with various components omitted (-) were used as controls.

**Figure S7.** *In vivo* activation of OsMPK3 and OsMPK6 by constitutively active OsMKK4.

Kinase activities in extracts of cells expressing *OsMKK4^WT^*, *OsMKK4^DD^*, *OsMKK5^WT^*, and *OsMKK5^DD^* were analyzed by an in-gel kinase assay using MBP as a substrate (each top panel). Each construct was under the control of a DEX-inducible promoter, and cells were treated with 10 μM DEX for the times indicated. As negative controls, cells transformed with the empty vector (Vec) were treated with DEX and with ethanol. The arrows indicate the position of OsMPK6 with the size of 45-kDa. Production of MAPKKs was confirmed by immuno-blot analysis using the anti-HA antibody (each middle panel). Equal loading was confirmed by CBB staining (each bottom panel).

**Figure S8.** Phenotypes of seedlings expressing *OsMKK4^DD^* or *OsMKK4^WT^*.

Plants transformed with the empty vector (Vec) were used as controls. Four-day-old seedlings grown without DEX were transferred to a medium containing 10 μM DEX. The seedlings were photographed at 0, 2, and 7 days after transfer. Two independent lines transformed with each construct had similar phenotypes.

**Figure S9.** Activation of kinases by the expression of OsM*KK4^DD^* in WT*, osmpk6,* and *osmpk6/OsMPK6* cells.

Kinase activities in extracts of cells expressing *OsMKK4^DD^* in WT*, osmpk6,* and *osmpk6/OsMPK6* cells were analyzed by an in-gel kinase assay using MBP as a substrate. *OsMKK4^DD^* was under the control of a DEX-inducible promoter, and cells were treated with 10 μM DEX for the times indicated. OsMPK6 and OsMKK4^DD^ were detected by immuno-blot analysis. Top panel indicates kinase activities. The arrows indicate the position of OsMPK6 with the size of 45-kDa and an unidentified protein band with the size of 43-kDa. OsMPK6 (2nd panel) and OsMKK4^DD^ (3rd panel) were detected by immuno-blot analysis. Equal loading was confirmed by CBB staining (bottom panel).

**Figure S10.** ROS and cell death assays in *Nicotiana benthamiana*.

*N. benthamiana* was infiltrated with *Agrobacterium* lines carrying *OsMKK4^WT^*, *OsMKK4^DD^* or *NtMEK2^DD^* and stained using DAB or trypan blue. Production of the MAPKKs was detected by immuno-blot analysis using the anti-HA antibody (upper left panel). Equal loading was confirmed by CBB staining (upper left panel). Position of inoculation in leaf was illustrated (upper right). Leaves were stained using DAB or trypan blue at 2 or 4 d post inoculation (right). Leaves were photographed before staining (left).

**Figure S11.** Summary of the genes regulated by OsMKK4^DD^.

Gene expression was compared in cells expressing the DEX-inducible *OsMKK4^DD^* construct and cells harboring the control vector, using Agilent rice 44K oligoarray with the two-color method. Cells expressing the DEX-inducible *OsMKK4^WT^* construct were also used as a control. Venn diagrams of the genes were drawn on the basis of three criteria: I. fold differences (OsMKK4^DD^ vs. vector control) at 12 h of DEX treatment were >2 or <0.5, II. ratio of fold differences (OsMKK4^DD^ vs. vector control) at 12 h and those at 0 h were >2 or <0.5, III. ratio of fold differences (OsMKK4^DD^ vs. vector control) at 12 h and those (OsMKK4^WT^ vs. vector control) at 12 h were >2 or <0.5.

**Figure S12.** Regulation of sugar metabolism pathway genes by Os MKK4^DD^.

The sugar metabolism pathway, showing enzymes whose genes were up- or down-regulated by OsMKK4^DD^ in WT cells. The numbers beside enzymes in the pathway indicate gene expression ratios (OsMKK4^DD^ vs. vector control) at 12 h after the beginning of DEX treatment. Red characters indicate up-regulation by OsMKK4^DD^. Asterisks show the significance of the differential expression according to the one sample *t*-test (*: P < 0.05. **: P < 0.01).

ADH, alcohol dehydrogenase; ALDH, aldehyde dehydrogenase; ALDP, fructose-bisphosphate aldolase; EN, enolase; FBPase, fructose-1,6-bisphosphatase; GAPDH, glyceraldehyde 3-phosphate dehydrogenase; GPI, glucose-6-phosphate isomerase; HK, hexokinase; PDC, pyruvate decarboxylase; PDH, pyruvate dehydrogenase; PFK, 6-phosphofructokinase; PFKB, fructokinase; PGAM, phosphoglycerate mutase; PGK, phosphoglycerate kinase; PK, Pyruvate kinase; SUS, sucrose synthase; TPP, trehalose phosphatase; TPS, trehalose phosphatase/synthase; TRE, trehalase; UDPGP, UTP-glucose-1-phosphate uridylyltransferase; VIN, vacuolar invertase.

**Figure S13.** Gene regulation in *osmpk6* cells expressing *OsMKK4^DD^*.

Effect of OsMPK6 on OsMKK4^DD^-induced gene expression was analyzed using the Agilent rice 44K oligoarray with the one-color method. Hierarchical clustering of the gene expression ratios for the samples comparing *osmpk6* and *osmpk6/OsMPK6* over DEX treatment (+) or control (ethanol) treatment (-). Similarity measurement was held by Pearson correlation using average linkage as a clustering algorithm. Each row represents a gene that was up or down regulated by OsMKK4^DD^ in WT cells at 12 h after the beginning of DEX treatment. Each column represents the mean of three biological replicates. Colors represent repression (blue) and induction (red) as indicated by the color bar. Reliable signals are shown in darker colors while unreliable signals are in paler colors. The heat maps are based on the same data as those in Figure 5B except that the values are relative to those in *osmpk6/OsMPK6* control samples.

**Figure S14.** Appearance of cells after OsMKK4^DD^ induction.

Appearance of cells treated for 3 d with 10 μM DEX, ethanol (control for DEX). Cells containing the DEX-inducible *OsMKK4^DD^* construct were used. Both WT and *osmpk6* cells expressing *OsMKK4^DD^* became brown three days after DEX treatment.

**Table S1.** List of genes significantly regulated by OsMKK4^DD^.

**Table S2.** Classification using Gene Ontology.

**Table S3.** List of genes related to sugar metabolism.

**Table S4.** Ribosomal protein genes.

**Table S5.** List of genes related to isoprenoid metabolism.

**Table S6.** List of genes related to phenylpropanoid metabolism.

**Appendix S1**

**Experimental procedures**

*Plant material*

Calli of *O. sativa* L. cv. Nipponbare were generated from seed scutella and cultured in N6D liquid suspension medium at 25˚C. The cells were sub-cultured in fresh medium every 7 days. Before elicitor, DPI, or DEX treatments, cells were sub-cultured for 3 days in fresh medium, then transferred to 60 mm dishes (0.4 g cells, 5 ml fresh medium), 35 mm dishes (0.1 g cells, 2 ml fresh medium) or 20 mm-well microplates (0.1 g cells, 1.5ml fresh medium per well), and maintained in suspension for 18 h at 25˚C with gentle shaking at 100 rpm. Incubations with shaking were continued for various lengths of time after treatments. Chitin elicitor (*N*-Acetylchitooctaose) was prepared by re-*N*-acetylation of the chitosan oligosaccharide (kindly supplied by Yaizu Suisan Kagaku Industrial Co. Ltd (Shizuoka, Japan)), and used at a final concentration of 1 μg/ml starting from a 0.5 mg/ml stock solution in water, except for the ROS assay (1 nM final concentration). Diphenyleneiodonium Choloride (DPI, Sigma) was used at a final concentration of 2 μM starting from a 10 mM stock solution in DMSO. Dexamethasone (DEX) was used at 10 μM, starting from a 10 mM stock solution in ethanol.

The *osmpk6* mutant (ND5549) was derived from a collection of rice mutant lines that were induced by insertion of the rice endogenous retrotransposon *Tos17* (Hirochika, 2001). For transformation of the *osmpk6* mutant, calli were made from segments of roots (T. Watanabe, K. Abe, R. Hirochika, A. M., K. Wakasa and H. H. unpublished data).

For DEX treatment of whole transgenic plants, seeds were sown under aseptic conditions on MS medium plates containing 0.2% gellangum, and grown at 25˚C. Four-day-old seedlings were transferred to MS medium containing 10 μM DEX, 3% sucrose and 0.2% gellangum, and grown at 25˚C.

*Rice transformation*

Agrobacterium-mediated transformation of rice calli was performed as described previously (Fu*se et a*l., 2001; Hi*ei et a*l., 1994). Plants were regenerated from transformed calli by selecting for hygromycin resistance.

*Plasmid construction*

The OsMPK6 complemented line (*osmpk6/OsMPK6*) was made using a construct containing the native OsMPK6 promoter and the ORF sequence of OsMPK6. The promoter (2.1 kb) was amplified from OSJNBa0085L11 (accession no. AP006533) using standard PCR methods with the primers OsMPK6proF (5’-AGTCCTCGAGTCCAAACTCTGCAGCAGGGT-3’) and OsMPK6UP (5’-GCCTGGATGTTCTCCATCAT-3’), and then digested with XhoI and NgoMIV. The cDNA was amplified using the primers OsMPK6PF (5’-ATGGACGCCGGGGCGCAGCC-3’) and OsMPK6PR (5’-ACTAGTCTACTGGTAATCAGGGTTGA-3’), and digested with NgoMIV and SpeI. The Nos terminator was isolated as a SpeI and SacI fragment from the plasmid pUCHindIIITnosSacI (M. Yamazaki and H. H., unpublished data, 2005). The promoter, cDNA, and Nos terminator fragments were ligated into the binary vector pPZP35sHPT-KS (H. Matsui, M. Yamazaki, M. K.-K., A. T. and H. H., in preparation), which was digested with XhoI and SacI to create pPZP35sHPT_OsMPK6proORFTNos.

The OsMPK3, OsMPK4, OsMPK6, OsMKK4 and OsMKK5 sequences (Os03g0285800, Os10g0533600, Os06g0154500, Os02g0787300 and Os06g0191300 (AK099769), respectively in the RAP database: <http://rapdb.dna.affrc.go.jp/>) were amplified by PCR. The sequences were subcloned into pENTR/D-TOPO (Invitrogen) and then into the pDEST17 bacterial expression vector to create His-tagged proteins (Invitrogen), using the LR clonase reaction. Mutant versions of the MAPK and MAPKK genes were generated by PCR using primers designed to create the amino acid conversions. The kinase inactive mutant of OsMPK6 (OsMPK6^KR^) was made by replacing the conserved Lys-96 in OsMPK6 with Arg. The constitutively active MAPKK mutants OsMKK4^DD^ and OsMKK5^DD^ were made by replacing conserved activation loop residues (Thr-239 and Ser-245 in OsMKK4; Thr-216 and Ser-222 in OsMKK5) with Asp.

To generate N-terminal HA-tagged sequences, the HA-His sequence was first amplified from p35S-ShΔ-dHA/His (Kagay*a et al*., 2002) using the primers HA_HisF1 (5’-ACTAGTATGAATTCCCGGGGATCCGTCG-3’), carrying the start codon and a SpeI recognition site; and HA_HisR (5’-GCGGCCGCGTGGTGGTGGTGGTGGTGAC-3’) carrying a NotI recognition site. The amplified fragment was used in a second round of PCR with the primers HA_HisR and HA_HisF2 (5’-GCGGCCGCTACTAGTATGAATTCCCGGGGA-3’), carrying a NotI recognition site. This amplified fragment was subcloned into the pCR4-TOPO vector (Invitrogen). The amplified HA-His sequence was digested with NotI and ligated into the NotI-digested pENTR/D-TOPO vector carrying OsMKK4 or OsMKK5. The HA-tagged MAPKKs were inserted downstream of the rice actin promoter in the binary vector pEASY-Act_pro_ (H Matsui, M Yamazaki, M. K.-K., A. T. and H. H., in preparation) using the LR clonase reaction, to generate constitutive expression lines. To generate DEX-inducible HA-tagged MAPKK expression lines, the pENTR/D-TOPO vector carrying HA-tagged OsMKK4 or OsMKK5 was digested with SpeI and BsrGI, and the digested fragments were inserted downstream of the 4UAS sequence in the binary vector pINDEX 2 (Ouwerker*k et al*., 2001). The plasmid carrying OsMKK4^DD^ under the control of the DEX inducible promoter was named pINDEX2_OsMKK4^DD^.

The cell line *osmpk6/OsMPK6*, expressing OsMKK4^DD^ under the control of the DEX inducible promoter, was made from pPZP35sHPT_OsMPK6proORFTNos and pINDEX2_OsMKK4^DD^. A fragment containing the promoter and ORF of OsMPK6 and the TNos terminator was amplified using standard PCR methods with the primers BstXI-OsMPK6pro (5’-CCACCATGTTGGTCCAAACTCTGCA-3’) and NosT-BstXI (5’-CCACCATGTTGGCCCGATCTAGTA-3’). The fragment was digested with BstXI and then ligated into the BstXI site of the binary vector pINDEX2.

*Antibody production and immuno-blot analyses*

Rabbit polyclonal antibodies against OsMPK3, OsMPK4 and OsMPK6 were developed using the following peptides as antigens: amino acids 1 to 15 of OsMPK3, amino acids 1 to 15 of OsMPK4, and amino acids 1 to 14 of OsMPK6. The antibodies were purified using the antigens. For immuno-blot analyses, the anti-OsMPK3 and anti-OsMPK4 antibodies were used in 1/3000 dilutions and the anti-OsMPK6 antibody was used in a 1/5000 dilution.

SDS-PAGE and protein gel blot analysis were performed as described previously (Takahash*i et al.*, 2007).

*Protein extraction, immuno-precipitation and MBP kinase assays*

Protein extractions and in gel kinase assays were performed as described previously (Kuru*su et a*l., 2005; Zhang and Klessig, 1997). Proteins were extracted from cells in extraction buffer (50 mM Hepes pH 7.4, 50 mM β-glycerophosphate, 5 mM EGTA, 5 mM EDTA, 10 mM NaF, 10 mM Na_3_VO_4_, 2 mM DTT, and Complete EDTA Free protease inhibitor cocktail (Roch)). The protein concentrations of the extracts were determined using the Bio-Rad protein assay kit with BSA as a standard. The extracts (10 μg proteins) were electrophoresed on 10% SDS-polyacrylamide gels embedded with 0.25 mg/ml myelin basic protein (MBP). The gels were dried on filter papers, and signals were visualized using a Bio Image Analyzer BAS2500 (Fuji-film, Tokyo, Japan).

For the MAPK IP kinase assays, 1 mg of proteins were pre-cleared overnight with 30 μl of protein-A-agarose (Roch) and then subjected to immunoprecipitation using 20 μl of protein-A-agarose and anti-MAPK antibody (5 μg for OsMPK3 and OsMPK4; 1 μg for OsMPK6) for 4 h at 4˚C. The beads were washed twice with wash buffer 1 (extraction buffer containing 150 mM NaCl, 1% Tween 20), twice with wash buffer 2 (extraction buffer, 500 mM NaCl, 0.1% Tween 20) and once with wash buffer 3 (extraction buffer, 0.1% Tween 20). The immunoprecipitated MAPKs were assayed for kinase activity in a reaction buffer (25 mM Tris-HCl (pH 7.5), 1 mM EGTA, 12 mM MgCl_2_, 1 mM DTT, 0.1 mM Na_3_VO_4_) containing 0.25 mg/ml MBP, 40 μM ATP and 1 μCi [γ-32P]ATP at 25˚C for 20 min. The reactions were stopped by the addition of Laemmli's sample buffer and boiling. Labeled proteins were analyzed by SDS-PAGE.

*Recombinant proteins and* in vitro *kinase assays*

MAPKs and MAPKKs were expressed as fusion proteins with an N-terminus poly-histidine tag using a bacterial expression system (Invitrogen) following the supplier’s instructions. The proteins were purified by immobilized metal ion affinity chromatography.

To detect the phosphorylation of MBP by recombinant MAPKs, 400 ng of each recombinant MAPK was pre-incubated for 15 min at 30˚C with 40 ng of MAPKK in a buffer containing 25 mM Tris-HCl (pH 7.5), 1 mM DTT, 1 mM EGTA, 10 mM MnCl_2_ and 50 μM ATP and then incubated with 1.5 μg MBP and 4 μCi [γ-^32^P]ATP for 30 min at 25˚C. The reaction was stopped by the addition of Laemmli's sample buffer and boiling. Labeled proteins were analyzed by SDS-PAGE.

*IP kinase assays of MAPKKs*

Protein extraction and IP kinase assays of MAPKKs were performed as described previously (Cardinal*e et al*., 2002). Proteins were extracted from cells expressing HA-tagged OsMKK4^WT^ and OsMKK5^WT^ constructs in MAPKK extraction buffer (25 mM Tris-HCl pH 7.5, 75 mM NaCl, 15 mM β-glycerophosphate, 15 mM EGTA, 10 mM MgCl_2_, 1 mM DTT, 1 mM NaF, 0.5 mM NaVO_3_, 15 mM *p*-nitrophenylphosphate, 0.1% Tween20, and Complete EDTA Free protease inhibitor cocktail (Roch)). The proteins (150 μg) were pre-cleared overnight with 10 μl of protein-G agarose (Roch) and then subjected to immunoprecipitation using 20 μl of protein-G agarose and 1 μl of anti-HA antibody (Covance) for 4 h at 4˚C. The beads were washed three times with a wash buffer (50 mM Tris-HCl pH 7.5, 250 mM NaCl, 5 mM EGTA, 5 mM EDTA and 0.1% Tween 20) and once with a kinase buffer (50 mM Tris-HCl pH 7.5, 1 mM DTT, 10 mM MgCl_2_). Kinase reactions were performed in the kinase buffer with 0.1 mM ATP, 1 μCi [γ-^32^P]ATP and 400 μg of OsMPK6^KR^ at 25˚C for 30 min. The reaction was stopped by the addition of Laemmli's sample buffer and boiling. Labeled proteins were analyzed by SDS-PAGE.

*RNA isolation and RT-PCR analysis*

Total RNA was isolated from rice cells using Isogen (Nippongene) and purified using an RNeasy for plants kit (Qiagen), according to the manufacturers’ protocols, and quantified with a spectrophotometer. First-strand cDNA was synthesized from 1 μg total RNA with an oligo-dT primer and reverse transcriptase (Toyobo). PCR amplification was performed using KOD-plus DNA polymerase (Toyobo). The OsMPK6 sequence was amplified using the primers OsMPK6_seqF (5’-GCATTGTCAGAGGAGCACTG-3’), and OsMPK6_seqR (5’- GAGGTGGTACGAGCAAGTCC-3’). The Actin sequence was amplified using the primers OsActF (5’-TGGAGAAGATCTGGCATCACAC-3’), and OsActR (5’-GACACCATCACCAGAGTCCAAC-3’).

*Microarray and data analysis*

For microarray data acquisition, Rice oligo microarrays (G2519F, Agilent Technologies) were hybridized according to the manufacturer’s instructions. Analyses were performed using RNA prepared from three independent experiments. RNA was fluorescence labeled according to the manufacturer’s protocol. The RNAs from WT cells expressing the *OsMKK4^DD^* or *OsMKK4^WT^* genes were labeled by with Cy3 and those from control cells harboring empty vector were labeled by with Cy5. One set of RNAs (OsMKK4^DD^ vs. vector control at 12 h after DEX treatment) was labeled by swapping the dyes (Cy3 and Cy5) to normalize for dye bias. The RNAs from *osmpk6* and *osmpk6/OsMPK6* background cells were labeled by with Cy3. Microarray slides were scanned using a microarray scanner (G2565B, Agilent), and resulting output files were imported into the Feature Extraction software (ver. 9.1; Agilent). Spot and background intensities from scanned slides were quantified, and data files were normalized, using the software algorithm Feature Extraction software. Data analysis was done performed using Genespring GX software (ver. 7.3; Agilent).

For the two-color method, the results were normalized compared the dye-swap data to eliminate the spots containing contradictory results between the two-color sets. Each probe’s measured intensity was divided by the vector control signal intensity in each sample; if the vector control signal intensity was below 10, then 10 was used instead. If both color signal intensities were below 10 then no data were reported. Signal intensities below 0.01 were set to 0.01. Each measurement was divided by the 50.0th percentile of all measurements in that sample to compensate for differences between slides. The normalized data were filtered with flag information to eliminate probe sets showing unreliable signal (absent on some slides) to identify candidate probe sets. We used a cross-gene error model to estimate population variance with a limited number of experiments. Statistical analysis of the normalized data was performed for the datasets of OsMKK4^DD^ and the vector control at each time point by means of a one sample *t*-test and correction for multiple testing using the false discovery rate (q < 0.1) (Storey and Tibshirani, 2003). Q-values were calculated using QVALUE software (ver. 1.0; <http://genomics.princeton.edu/storeylab/qvalue/>).

For the one-color method, the results were normalized with two steps: signal intensities below 0.01 were set to 0.01, then each measurement was divided by the 50.0th percentile of all measurements in that sample to compensate for differences between slides.

Data from the selected probe sets were imported into the Gene ontology browser using the Genespring GX software algorithm. To search the genes encoding the enzymes in the metabolic pathways for sugar, aromatic compounds and isoprenoids, The Kyoto Encyclopedia of Genes and Genomes (KEGG) database (<http://www.kegg.jp/ja/>) (Kanehi*sa et a*l., 2008; Oga*ta et a*l., 1999) was used to search for genes encoding enzymes in the metabolic pathways for sugar, aromatic compounds and isoprenoids. The Salad database (<http://salad.dna.affrc.go.jp/salad/>) (Mihar*a et al*., 2008) was used to search for structurally related genes from Arabidopsis and rice. The Plant Transcription Factor Database (PlnTFDB ver.2.0; http://plntfdb.bio.uni-potsdam.de/) (Riano-Pacho*n et al*., 2007) was used to classify transcription factors.

*Selection of genes regulated by OsMKK4^DD^ in WT cells*

Gene expression was compared in WT cells expressing the DEX-inducible OsMKK4^DD^ construct and cells harboring the control vector, using the Agilent rice 44K oligoarray with the two-color method. WT cells expressing the DEX-inducible OsMKK4^WT^ construct were also used as a control. We chose time points of 0, 2, 6**,** and 12 h after DEX treatment for sampling. Statistical analyses of the normalized data were performed between the data sets for OsMKK4^DD^ and the vector control at each time point, by means of a one sample *t*-test and correction for multiple testing using the false discovery rate (q < 0.1) (Storey and Tibshirani, 2003). Differential expression was detected for 9,540 genes at 12 h after the onset of DEX treatment. Genes that satisfied the following three criteria were selected for further analysis: I. fold differences (OsMKK4^DD^ vs. vector control) at 12 h of DEX treatment were >2 or <0.5, II. ratio of fold differences (OsMKK4^DD^ vs. vector control) at 12 h and those at 0 h were >2 or <0.5, III. numbers of genes with altered expression in the data set comparing OsMKK4^DD^ vs. vector control at 12 h with OsMKK4^WT^ vs. vector control at 12 h (Figure S11, Table S1).

*Agroinfiltration of* N. benthamiana

Agroinfiltration of *N. benthamiana* was performed as described previously (Kobayashi et al., 2007)**,** with modifications. Binary vector constructs were made by inserting DNA fragments of the control GUS gene, HA:NtMEK2^DD^ (Takabatak*e et al*., 2007), HA:OsMKK4^WT^, or HA:OsMKK4^DD^ into the binary vector pEl2Ω (Mitsuhar*a et al.*, 1996). Transgenic lines of *Rhizobium radiobacter* (*Agrobacterium tumefaciens*) strain EHA105 carrying the binary constructs were used to infiltrate 5-week old *N. benthamiana* leaves. Each *Agrobacterium* culture was grown to an OD600 of 0.5 then resuspended in a buffer containing 10 mM MgCl_2_, 10 mM MES, pH 5.6, and 100 μM acetosyringone. The suspensions were incubated at 23˚C for 4–5 h and diluted to an OD600 of 0.2 in the same buffer**,** before infiltrating into the fourth and fifth leaves of *N. benthaniana* using a needleless 1-mL syringe. The agroinfiltrated plants were kept in a growth chamber at 75% humidity for 4 days after agroinfiltration.

Expression of HA-tagged MAPKKs was confirmed by immunoblot analysis using an anti-HA antibody. The blot was also stained with CBB. To visualize ROS *in situ*, DAB staining was performed as described previously (Thordal-Christense*n et al.*, 1997). The agroinfiltrated leaves were detached and incubated in 1 mg/ml DAB solution for 8 h. Then, the leaves were fixed with a solution of 3:1 ethanol/acetic acid. The HR was detected by trypan blue staining as described (Koch and Slusarenko, 1990).

# References

**Cardinale, F., Meskiene, I., Ouaked, F. and Hirt, H.** (2002) Convergence and divergence of stress-induced mitogen-activated protein kinase signaling pathways at the level of two distinct mitogen-activated protein kinase kinases. *Plant Cell*, **14**, 703-711.

**Fuse, T., Sasaki, T. and Yano, M.** (2001) Ti-plasmid vectors useful for functional analysis of rice genes. *Plant Biotechnol*, **18**, 219-222.

**Hiei, Y., Ohta, S., Komari, T. and Kumashiro, T.** (1994) Efficient transformation of rice (Oryza sativa L.) mediated by Agrobacterium and sequence analysis of the boundaries of the T-DNA. *Plant J.*, **6**, 271-282.

**Hirochika, H.** (2001) Contribution of the Tos17 retrotransposon to rice functional genomics. *Curr. Opin. Plant Biol.*, **4**, 118-122.

**Kagaya, Y., Hobo, T., Murata, M., Ban, A. and Hattori, T.** (2002) Abscisic acid-induced transcription is mediated by phosphorylation of an abscisic acid response element binding factor, TRAB1. *Plant Cell*, **14**, 3177-3189.

**Kanehisa, M., Araki, M., Goto, S., Hattori, M., Hirakawa, M., Itoh, M., Katayama, T., Kawashima, S., Okuda, S., Tokimatsu, T. and Yamanishi, Y.** (2008) KEGG for linking genomes to life and the environment. *Nucleic Acids Res*, **36**, D480-484.

**Kobayashi, M., Ohura, I., Kawakita, K., Yokota, N., Fujiwara, M., Shimamoto, K., Doke, N. and Yoshioka, H.** (2007) Calcium-dependent protein kinases regulate the production of reactive oxygen species by potato NADPH oxidase. *Plant Cell*, **19**, 1065-1080.

**Koch, E. and Slusarenko, A.** (1990) Arabidopsis is susceptible to infection by a downy mildew fungus. *Plant Cell*, **2**, 437-445.

**Kurusu, T., Yagala, T., Miyao, A., Hirochika, H. and Kuchitsu, K.** (2005) Identification of a putative voltage-gated Ca2+ channel as a key regulator of elicitor-induced hypersensitive cell death and mitogen-activated protein kinase activation in rice. *Plant J.*, **42**, 798-809.

**Mihara, M., Itoh, T. and Izawa, T.** (2008) In silico identification of short nucleotide sequences associated with gene expression of pollen development in rice. *Plant Cell Physiol.*, **49**, 1451-1464.

**Mitsuhara, I., Ugaki, M., Hirochika, H., Ohshima, M., Murakami, T., Gotoh, Y., Katayose, Y., Nakamura, S., Honkura, R., Nishimiya, S., Ueno, K., Mochizuki, A., Tanimoto, H., Tsugawa, H., Otsuki, Y. and Ohashi, Y.** (1996) Efficient promoter cassettes for enhanced expression of foreign genes in dicotyledonous and monocotyledonous plants. *Plant Cell Physiol.*, **37**, 49-59.

**Ogata, H., Goto, S., Sato, K., Fujibuchi, W., Bono, H. and Kanehisa, M.** (1999) KEGG: Kyoto Encyclopedia of Genes and Genomes. *Nucleic Acids Res*, **27**, 29-34.

**Ouwerkerk, P.B., de Kam, R.J., Hoge, J.H. and Meijer, A.H.** (2001) Glucocorticoid-inducible gene expression in rice. *Planta*, **213**, 370-378.

**Riano-Pachon, D.M., Ruzicic, S., Dreyer, I. and Mueller-Roeber, B.** (2007) PlnTFDB: an integrative plant transcription factor database. *BMC Bioinformatics*, **8**, 42.

**Storey, J.D. and Tibshirani, R.** (2003) Statistical significance for genomewide studies. *Proc. Natl. Acad. Sci. U. S. A.*, **100**, 9440-9445.

**Takabatake, R., Ando, Y., Seo, S., Katou, S., Tsuda, S., Ohashi, Y. and Mitsuhara, I.** (2007) MAP kinases function downstream of HSP90 and upstream of mitochondria in TMV resistance gene N-mediated hypersensitive cell death. *Plant Cell Physiol.*, **48**, 498-510.

**Takahashi, A., Agrawal, G.K., Yamazaki, M., Onosato, K., Miyao, A., Kawasaki, T., Shimamoto, K. and Hirochika, H.** (2007) Rice Pti1a negatively regulates RAR1-dependent defense responses. *Plant Cell*, **19**, 2940-2951.

**Thordal-Christensen, H., Zhang, Z., Wei, Y. and Collinge, D.B.** (1997) Subcellular localization of H2O2 in plants. H2O2 accumulation in papillae and hypersensitive response during the barley—powdery mildew interaction. *Plant J.*, **11**, 1187-1194.

**Zhang, S. and Klessig, D.F.** (1997) Salicylic acid activates a 48-kD MAP kinase in tobacco. *Plant Cell*, **9**, 809-824.
